# Multiplexed relative and absolute quantitative immunopeptidomics reveals MHC I repertoire alterations induced by CDK4/6 inhibition

**DOI:** 10.1038/s41467-020-16588-9

**Published:** 2020-06-02

**Authors:** Lauren E. Stopfer, Joshua M. Mesfin, Brian A. Joughin, Douglas A. Lauffenburger, Forest M. White

**Affiliations:** 1 Koch Institute for Integrative Cancer Research, Massachusetts Institute of Technology, Cambridge, MA 02142 USA; 2 Department of Biological Engineering, Massachusetts Institute of Technology, Cambridge, MA 02139 USA

**Keywords:** Peptides, Mass spectrometry, Proteomics, Melanoma, MHC class I

## Abstract

Peptides bound to class I major histocompatibility complexes (MHC) play a critical role in immune cell recognition and can trigger an antitumor immune response in cancer. Surface MHC levels can be modulated by anticancer agents, altering immunity. However, understanding the peptide repertoire’s response to treatment remains challenging and is limited by quantitative mass spectrometry-based strategies lacking normalization controls. We describe an experimental platform that leverages recombinant heavy isotope-coded peptide MHCs (hipMHCs) and multiplex isotope tagging to quantify peptide repertoire alterations using low sample input. HipMHCs improve quantitative accuracy of peptide repertoire changes by normalizing for variation across analyses and enable absolute quantification using internal calibrants to determine copies per cell of MHC antigens, which can inform immunotherapy design. Applying this platform in melanoma cell lines to profile the immunopeptidome response to CDK4/6 inhibition and interferon-γ — known modulators of antigen presentation — uncovers treatment-specific alterations, connecting the intracellular response to extracellular immune presentation.

## Abstract (web summary)

Immunopeptidomics allows identifying the cellular repertoire of MHC-bound peptides, but quantifying them remains challenging. Here, the authors present a method to efficiently generate internal peptide MHC standards and calibration curves, facilitating relative and absolute quantitative immunopeptidomics.

## Introduction

Cells present signals on the extracellular surface that serve as targets for immune cell recognition. These signals, peptides presented by class I major histocompatibility complexes (MHCs), are typically derived from intracellular source proteins, and may therefore provide an external representation of the internal cell state^1^. As a reflection of this, the peptide MHC (pMHC) repertoire, or “immunopeptidome”, of cancer cells may contain tumor-associated or mutation-containing antigens that serve as tumor-specific markers to activate T cells and initiate an antitumor immune response. This interaction can be strengthened with checkpoint blockade (CB) immunotherapies; however, low response rates and toxicity remain barriers to their broad clinical success^2,3^. A growing body of evidence suggests that combining CB with other treatments, such as small-molecule inhibitors, cytotoxic agents, and radiotherapy, could potentiate the response to CB, in part, by augmenting tumor immunogenicity through increased surface pMHC expression^4–7^. While clinical trials in this space have shown promise^8,9^, the optimal combination of agents, as well as the order and timing of administration, are only beginning to be understood. In order to improve combinatorial strategies, a quantitative, molecular understanding of how different perturbations shift the immunopeptidome is required. Furthermore, achieving absolute quantification of presented antigens is necessary to inform immunotherapy drug design, as targeted strategies have varying thresholds of antigen expression required for an optimal antitumor response.

Traditional data-dependent acquisition (DDA) methods to profile pMHC repertoires using mass spectrometry (MS) are well documented^10–12^, but quantitative methods have critical limitations. Specifically, most common relative quantification pMHC methods lack a normalization strategy to account for variations in sample input and processing^13–18^. Peptide losses during processing vary across peptide sequences, concentrations, and samples, underscoring the need for normalization^19,20^. Absolute quantification of pMHCs to date is most commonly performed by comparing endogenous levels of pMHCs to exogenous peptide standards, again failing to account for sample losses^21–24^. Losses can be accounted for with internal pMHC standards, but require laborious refolding of pMHCs for every target of interest^19,25^. Nevertheless, this approach relies on single point calibration, ignoring the effects of ion suppression, thereby inaccurately estimating absolute pMHC levels in quantitative analyses.

To combat these challenges in quantitative immunopeptidomic profiling, we present a platform that utilizes ultraviolet (UV)-mediated peptide exchange of recombinant MHC monomers to generate on demand heavy-isotope-labeled pMHCs for relative and absolute quantification of pMHC repertoires using low sample input. We demonstrate that the addition of heavy-isotope pMHCs (hipMHCs) spiked into sample lysates for normalization improves quantitative accuracy between samples for both label-free (LF) and multiplexed (tandem mass tags (TMTs) labeled) analyses and provides an estimate of ion suppression through regression against a titrated internal calibrant. Furthermore, we utilize hipMHC multipoint-embedded standard curves coupled with isobaric mass tags to accurately quantify the absolute number of copies per cell of target antigens within a single analysis. We apply this platform to profile immunopeptidomic changes in melanoma cell lines, comparing treatment with palbociclib (a small-molecule CDK4/6 inhibitor) and interferon-γ (IFN-γ), both known modulators of antigen presentation^7,26^. Peptides derived from proteins implicated in the biological response to palbociclib and IFN-γ are selectively enriched in the pMHC repertoire following treatment, connecting the intracellular response to extracellular immune presentation. Furthermore, peptides derived from the metabolic response to palbociclib, along with known tumor-associated antigens (TAAs), display significantly increased presentation with palbociclib treatment. We propose this platform can be broadly applied to profile immunopeptidomic changes in a high-throughput, low-input format across sample types and treatments to inform combination therapy strategies and can be used to identify and quantify treatment-modulated antigen targets for targeted immunotherapy.

## Results

### Platform for relative and absolute pMHC quantitation

**Fig. 1:**
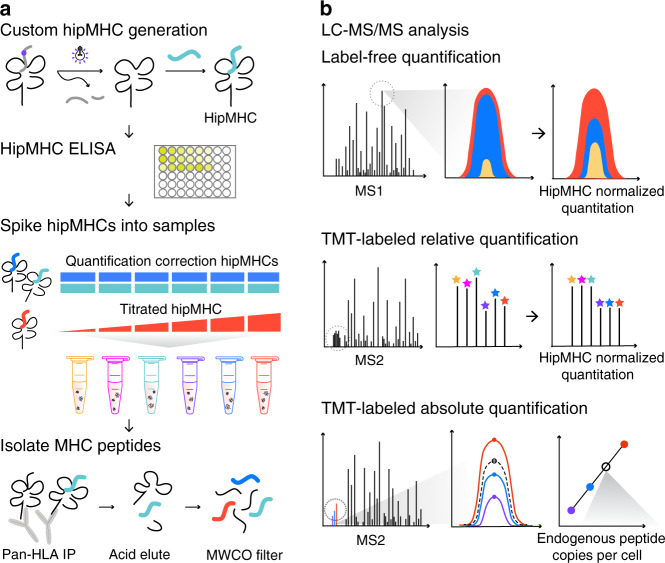
Platform for quantitative immunopeptidomics using hipMHCs. **a** HipMHCs were generated through UV-mediated peptide exchange of HLA*A2:01 monomers with a heavy leucine HLA-A*02:01 binding peptide. Stable hipMHCs concentrations were measured with an ELISA, and hipMHC complexes were added to lysate samples prior to immunoprecipitation (IP), at the same concentration for quantification correction (blue/teal) or titrated in to create an internal standard curve (red). Heavy and light pMHCs were isolated with IP, acid elution, and molecular weight cut-off (MWCO) filters. **b** Peptides were analyzed by LC-MS/MS three ways. Relative quantification label-free analyses were quantified by integrating the area under the curve (AUC) of the chromatographic elution across samples, and quantification was normalized by applying correction factors determined by hipMHC AUC intensity ratios between samples. Samples for multiplexed analysis were TMT-labeled and relative quantification was implemented using reporter ion intensities. Normalization was performed using hipMHC reporter ion intensity ratios across TMT channels. For absolute quantification, TMT-labeled samples containing a hipMHC internal standard curve were used to calculate the endogenous copies per cell of the pMHC of interest.

We set out to develop a platform to provide accurate relative and absolute quantification of pMHCs across multiple samples while controlling for losses associated with sample processing and enrichment. Accurate quantitative analysis is best performed with internal standards and multipoint internal calibration curves. To generate internal standards, heavy-isotope-labeled MHC peptides of interest were synthesized and loaded onto biotinylated MHC monomers through UV-mediated peptide exchange^27^ (Fig. 1a). To control for loading efficiency of synthetic peptides into recombinant MHC proteins, the concentration of stable hipMHC complexes was determined by an enzyme-linked immunosorbent assay (ELISA). Stable hipMHC complexes were then used in two ways: selected hipMHC complexes were spiked at the same concentration into the whole-cell lysate from each sample to provide a normalization correction for relative quantification across samples, while other hipMHC complexes were titrated at different concentrations into each sample to verify correction parameters, estimate dynamic range suppression for quantification, and/or create an internal standard curve for absolute quantification of a specific peptide. After adding hipMHCs, endogenous and exogenous pMHCs were isolated by immunoprecipitation (IP), acid elution, and molecular weight size-exclusion filtration.

Peptide mixtures were next analyzed by liquid chromatography-tandem mass spectrometry (LC-MS/MS) in three different ways (Fig. 1b). For LF analyses, samples were analyzed individually, and peptides were quantified by integrating the area under the curve (AUC) for the chromatographic elution of precursor masses for each peptide-spectrum match (PSM). Relative AUC intensities of quantification correction hipMHCs were used to normalize AUC intensities of endogenous peptides across analyses. To analyze multiple samples simultaneously, we labeled samples with TMT and relative TMT ion intensity ratios of hipMHCs were used for normalization to correct the relative quantification in multiplexed samples. TMT-labeled titrated hipMHCs were also used for absolute quantification of endogenous peptides. Apex TMT intensities of hipMHCs generated a peptide-specific multipoint calibration curve to calculate the average number of copies per cell. As a control, heavy-isotope-coded synthetic peptides not complexed to MHCs were spiked into the whole-cell lysate prior to IP. These peptides were not detected in the subsequent LC-MS/MS analysis, demonstrating that only peptides in stable complexes were isolated in our workflow and that excess free peptides did not displace endogenously presented peptides.

### HipMHC standards improve quantitative accuracy

**Fig. 2:**
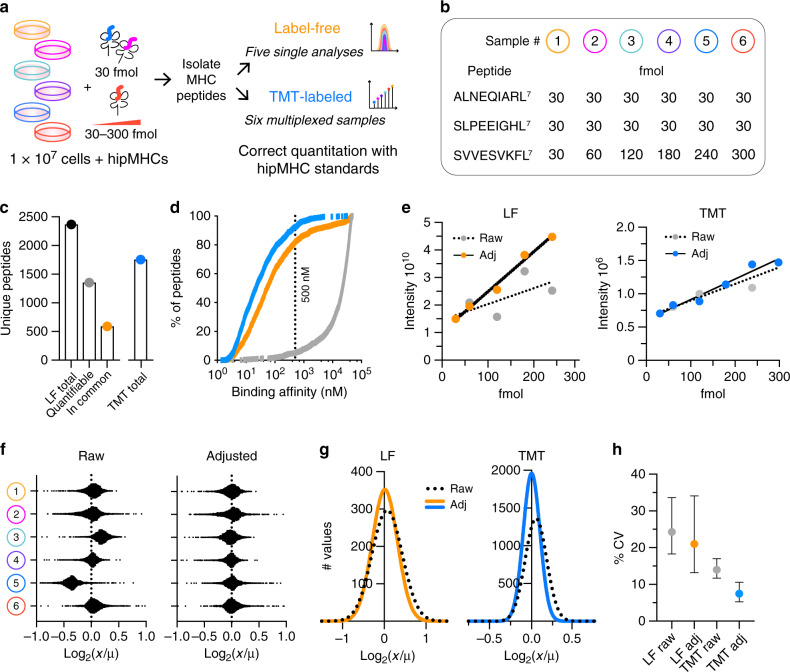
HipMHCs improve quantitation in LF and TMT-labeled samples. **a** Experimental design. Five LF (orange) and six TMT (blue) technical replicates of 1 × 10^7^ cells + hipMHCs were used to compare LF and TMT quantification. **b** Peptide sequence and amount of hipMHC added into each sample. L^7^ denotes heavy-isotope-labeled leucine (+7). ALNEQIARL^7^ and SLEEPIGHL^7^ were used as quantification correction hipMHCs, and SVVESVKFL^7^ was titrated in across samples. For LF analysis, sample #6 was omitted. **c** Two thousand three hundred and sixty-nine unique LF peptides were identified across five analyses (black), 1352 of which were quantifiable (gray) via AUC quantification, and 589 quantifiable peptides which were identified in all five analyses (orange). One thousand seven hundred and fifty-four unique peptides were quantified with TMT-labeled analyses combining six TMT fractions (blue). **d** Ninety-two percent of TMT, 82% of LF, and 5.6% of random peptide 9-mers derived from the proteome (gray) are predicted to bind to an HLA allele in MDA-MB-231 cells with an affinity < 500 nM. **e** Linear fit of titrated hipMHC peptide for LF (left) and TMT (right). Raw *r*^2^ = 0.48 (LF) and *r*^2^ = 0.91 (TMT), hipMHC-adjusted (adj) *r*^2^ = 0.99 (LF) and *r*^2^ = 0.96 (TMT). **f** Distribution of the log_2_ fold change (FC) of each peptide’s quantification (*x*) over the mean (*μ*) peptide quantification across samples for raw (left) and hipMHC adjusted (right). **g** Gaussian fit of the frequency distribution of log_2_(FC) of (*x*) over (*μ*) for raw and hipMHC-adjusted LF and TMT samples. In all, 99.7% of variance between peptide quantitation (3× SD) is captured within a 1.65 (raw) and 1.52 (adj) FC from the mean for LF samples, and 1.30 (raw) and 1.23 (adj.) for TMT samples. **h** Median coefficient of variation (CV) for LF (24.27% raw, 20.99% adj) and TMT (14.00% raw, 7.48% adj). Error bars represent the interquartile range. Source data are provided in the Source data file.

To demonstrate the improved quantitative accuracy obtained with hipMHCs, we used five LF and six TMT-labeled technical replicates of 1 × 10^7^ MDA-MB-231 breast cancer cells to measure variance between replicates before and after hipMHC correction (Fig. 2a, Supplementary Data 1). In both LF and TMT-labeled workflows, we spiked 30 fmol of two quantification correction hipMHCs into each sample, as adding additional correction hipMHCs gave minimal improvement in quantitative accuracy. We also added 30–300 fmol of a titrated hipMHC across samples (Fig. 2b). A total of 2369 unique pMHCs were identified in total across five LF analyses, 1352 of which were quantifiable via AUC integration (Fig. 2c). Of these quantifiable peptides, only 589 were quantified in all five analyses, highlighting the poor run-to-run overlap of LF analyses, even with replicate samples (Supplementary Fig. 1a). By comparison, 1754 unique peptides were quantifiable with TMT-labeled analyses. The extra sample handling steps associated with TMT labeling can result in losses, so to achieve high coverage of the immunopeptidome, labeled samples were divided into six separate analyses, thereby increasing the number of unique identifications (Supplementary Fig. 1b). In both LF and TMT analyses, peptides matched expected length distributions of 8–11 amino acids (Supplementary Fig. 1c), and 82% of LF and 92% of TMT 9-mers were predicted to be binders with <500 nM predicted affinity^28^ (Fig. 2d). The peptides were similarly apportioned across alleles between LF and TMT analyses, and the allele motifs aligned with those previously reported^29^ (Supplementary Fig. 1d, e). Further reducing the input material to 5 × 10^6^ cells still resulted in 86% of the number of unique peptides identified with 1 × 10^7^ cells in a single LF analysis, establishing the sensitivity of this method for low-input pMHC analyses (Supplementary Fig. 1f).

To normalize LF and TMT-labeled data sets, we applied correction parameters calculated from the quantification correction hipMHCs. The titrated peptide, SVVESVKFL^7^, displayed an improved linear fit after correction, with an even more pronounced effect in the LF samples (Fig. 2e). We observed dynamic range suppression for this peptide in TMT-labeled (4.7×) and LF (2.7×) data sets, demonstrating in both cases that quantitative differences are likely larger than what is measured.

In both analyses, hipMHC quantification correction reduced variation, for example, peptides from TMT-labeled sample 5 have lower intensities than the other samples, which was corrected by hipMHC normalization (Fig. 2f). The standard deviation from the mean for replicate samples decreased with hipMHC correction in LF and TMT-labeled samples (Fig. 2g), although TMT labeling showed lower variation between replicates (Fig. 2h), allowing for higher confidence in small shifts within the immunopeptidome. We investigated whether peptides with lower abundance had higher quantitative variation across samples, but found no correlation in LF or TMT-labeled analyses (Supplementary Fig. 1g).

### Absolute quantification of endogenous pMHCs

**Fig. 3:**
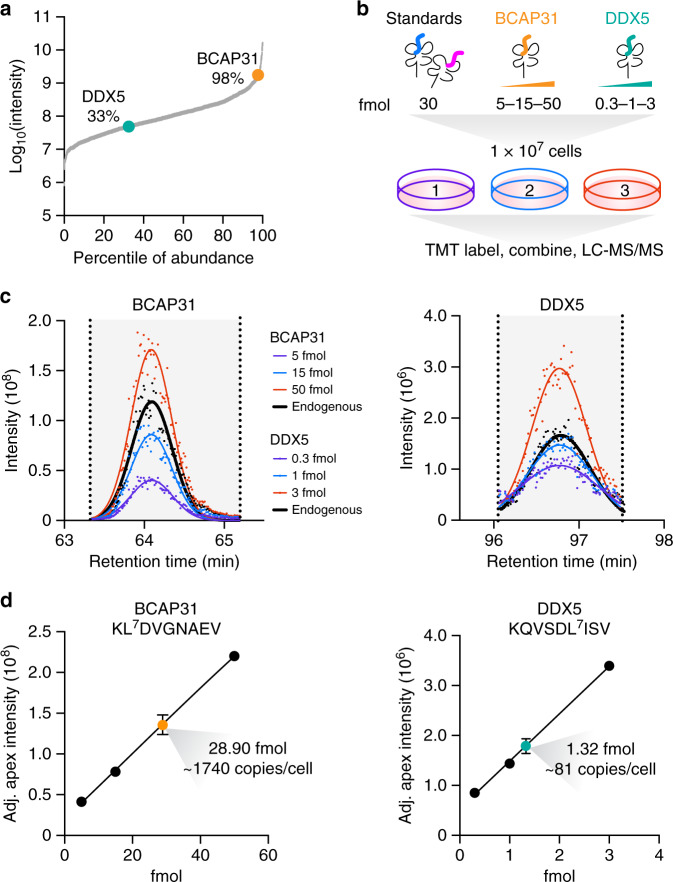
Absolute quantification of pMHCs with hipMHC standards and isobaric labeling. **a** Peptide intensities for TMT-labeled MDA-MB-231 cells from Fig. 2 were determined by AUC quantification. Percentile of abundance represents a peptide’s rank relative to the most abundant peptide. **b** Experimental design. Normalization standards along with 5, 15, and 50 fmol of BCAP31 and 0.3, 1, and 3 fmol of DDX5 hipMHCs were added to three biological replicates of 1 × 10^7^ MDA-MB-231 cells, and peptides from MHC complexes were isolated, labeled, and analyzed via LC-MS/MS. **c** Chromatographic elution profiles for the three TMT reporter ion intensities of the hipMHC standard curve (colored), along with the average (*n* = 3) TMT reporter ion intensity trace of the endogenous peptide (black). Each MS2 scan is represented as a single point, and elution profiles are fitted with a gaussian distribution (line). **d** Adjusted (hipMHC normalized) apex intensity versus fmol of hipMHC added creates a standard curve from which the endogenous concentration of antigen is calculated. For both linear fits of BCAP31 and DDX5, *r*^2^ > 0.999. The endogenous peptide is presented as the mean value ± SD for *n* = 3 biological replicates. Source data are provided in the Source data file.

To demonstrate the ability of hipMHCs to quantify pMHC copies per cell, we selected two peptides identified in TMT-labeled MDA-MB-231 cells for absolute quantification: KLDVGNAEV derived from B cell receptor-associated protein 31 (BCAP31) and KQVSDLISV from DEAD-box RNA helicase 5 (DDX5). BCAP31 regulates the transport of membrane proteins from the endoplasmic reticulum to the Golgi, a central component of antigen processing, and is a known TAA peptide^30^. DDX5 is important in gene expression regulation and has been implicated in proliferation, metastasis, and tumorigenesis in cancer^31^. These peptides were detected at differing levels with the highly abundant BCAP31 peptide falling in the 98th percentile and DDX5 falling in the 33rd percentile of abundance (Fig. 3a). Both peptides were synthesized with heavy-isotope-labeled leucine (+7), and hipMHC normalization standards were added to three replicates of 1 × 10^7^ MDA-MB-231 cells along with titrated amounts of BCAP31 and DDX5 hipMHC (Fig. 3b). We then labeled samples with TMT and performed LC-MS/MS analysis using an inclusion list, so only targeted peptides of interest were selected for fragmentation (Supplementary Data 2).

Chromatographic traces of the three TMT reporter ions for heavy BCAP31 and DDX5 peptides displayed increasing ion intensities with increasing amount of hipMHC added (Fig. 3c). In order to quantify peptide expression, the apex intensities of reporter ions were adjusted based on the normalization hipMHCs, and a linear fit was used to determine pMHC concentration present in the sample. Cells had an average of 1740 copies per cell of the BCAP31 peptide, and 81 copies per cell of the DDX5 peptide (Fig. 3d). Concentrations of the DDX5 hipMHC as low as 100 attomole were detected (six copies per cell) showcasing the broad range of pMHC expression levels quantifiable by our method (Supplementary Fig. 2). Furthermore, BCAP31 and DDX5 had a dynamic range suppression of 1.9× and 2.5×, respectively, illustrating that the ion suppression is not uniform across peptides and that peptide-specific internal standards may be required for absolute quantification of each pMHC of interest.

### CDK4/6 inhibition alters the pMHC repertoire in melanoma

Cyclin-dependent kinases 4 and 6 (CDK4/6) control cell cycle progression by phosphorylating Rb1, thereby releasing the E2F family of transcription factors that drive progression through the G1 checkpoint^32^. CDK4/6 is often dysregulated and overactive in cancer, leading to uncontrolled proliferation^33^. As such, CDK4/6 inhibitors have emerged as a potentially powerful class of anticancer agents, active against a spectrum of tumor types including melanoma^34^. In recent years, CDK4/6 inhibitors have also been shown to enhance tumor immunogenicity by increasing surface MHC class I expression and boosting T cell activation and infiltration^7,35^. These data highlight CDK4/6 inhibitors as an attractive candidate to combine with CB or other immunotherapies to augment immunotherapy response rates in melanoma. However, to date, the effect of CDK4/6 inhibition on the MHC class I peptide repertoire has not been characterized. We therefore applied our platform to quantify how pMHC repertoires in melanoma change in vitro upon treatment with the CDK4/6 inhibitor, palbociclib, to better understand how CDK4/6 inhibitors could be leveraged in combination therapy regimes to improve patient outcomes.

**Fig. 4:**
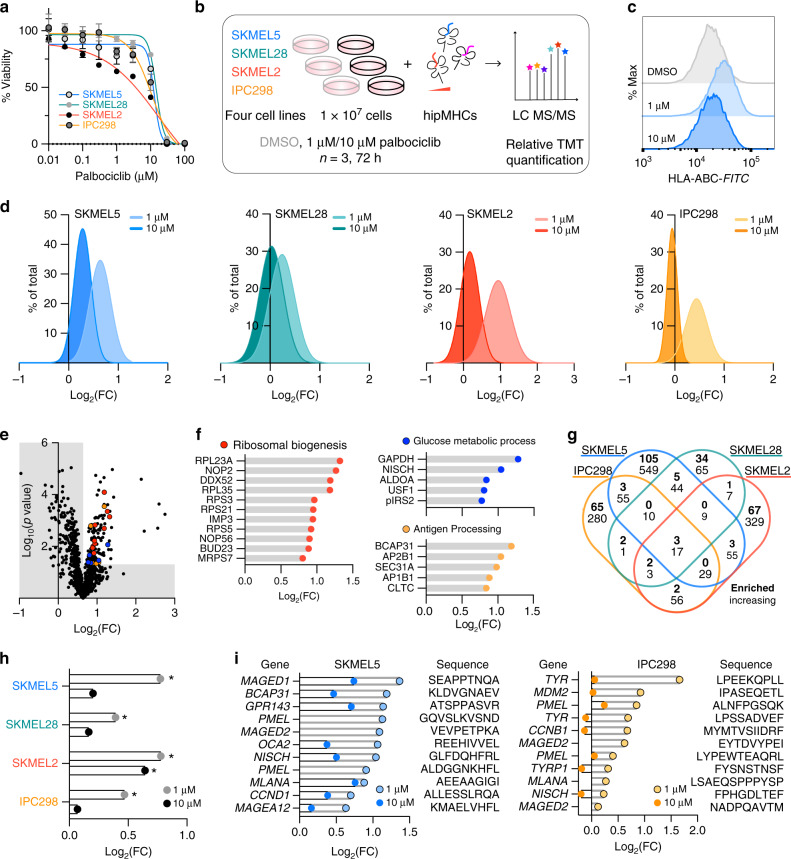
Palbociclib alters the immunopeptidome in melanoma. **a** Viability at 72 h after drug treatment; data are represented as a fraction (%) of the DMSO control. Calculated IC_50_s: SKMEL5 = 12.74 μM, SKMEL28 = 14.62 μM, SKMEL2 = 16.98 μM, and IPC298 = 10.62 μM. Data are presented as mean values ± SD for *n* = 3 experimental replicates for all cell lines, except SKMEL5 (*n* = 4). **b** Experimental setup of TMT-labeled immunopeptidomics experiments in melanoma cell lines. **c** Flow cytometry measurements of surface HLA expression in SKMEL5 cells. Data are represented as % of maximum signal, and the distributions are representative of three independent experiments. **d** Histogram distribution of log_2_ fold change (FC) of (palbociclib/DMSO) for unique pMHCs, where FC is calculated from the mean intensity of *n* = 3 biological replicates per condition. Data are represented as a % of total unique peptides identified. **e** Volcano plot displaying log_2_(FC) of 1 μM treated SKMEL5 cells versus significance (mean-adjusted *p* value, unpaired two-sided *t* test). Colored points (*p* < 0.05, log_2_(FC) > 1.56) correspond to processes in **f**. **f** Log_2_(FC) of significantly enriched peptides from GO term enrichment processes labeled with source protein name. False discovery rate (FDR)-adj. *p* value < 0.05. **g** Four-way Venn diagram of the number of source proteins of peptides significantly enriched (top value) or significantly increasing (bottom value) with 1 μM palbociclib. **h** Log_2_(FC) of pIRS2 peptide following 1 μM (gray) or 10 μM (black) palbociclib, *p < 0.05, unpaired two-sided *t* test. **i** Source protein name, peptide sequence, and log_2_(FC) of TAAs in SKMEL5 (left) and IPC298 (right) cells. Exact significance values and other source data are reported in the Source Data File.

We selected four melanoma cell lines for analysis: SKMEL5 and SKMEL28 (BRAF mutant), and SKMEL2 and IPC298 (NRAS mutant). Based on sensitivity analyses for each cell line (Fig. 4a), we selected two doses of palbociclib for further study: a low dose of 1 μM, below the half-maximal inhibitory concentrations (IC_50_) of all four cell lines, and a high dose of 10 μM, near the IC_50_. Three biological replicates of 1 × 10^7^ cells of each cell line were then treated with dimethyl sulfoxide (DMSO), and low- or high-dose palbociclib for 72 h (Fig. 4b). Low-dose treatment increased surface class I MHC presentation, as measured by flow cytometry, by 1.5–2× across cell lines, whereas high-dose treatment had a milder effect (Fig. 4c, Supplementary Fig. 3a).

To characterize the pMHC repertoire alterations induced by palbociclib, multiplexed relative quantitation was performed comparing low- and high-dose palbociclib to DMSO for each cell line, and data were normalized using hipMHC standards (Supplementary Fig. 3b, Supplementary Data 3). As with our previous analysis, identified peptides matched expected length distributions, and a majority were predicted to be MHC class I binders (Supplementary Fig. 3c, d). Immunopeptidomic analysis for each cell line and treatment showed a similar trend to the flow cytometry data: low-dose palbociclib shifted mean pMHC expression higher than DMSO treatment in all cell lines, and a high-dose palbociclib showed a small increase in mean expression for SKMEL5 compared to DMSO and no significant change for the other cell lines (Fig. 4d, Supplementary Fig. 3e). We measured a wider distribution of changes in peptide presentation following low-dose treatment, with several peptides increasing eight- to ten-fold, even before considering the effect of dynamic range suppression (Supplementary Fig. 3b).

To gain insight into the biology underlying palbociclib-modulated pMHC alterations, we analyzed our data in two ways. First, we determined which peptides and source proteins were significantly increased with palbociclib treatment over DMSO. Because many peptides were significantly increased with low-dose treatment, we also identified the peptides and source proteins that were significantly enriched in presentation with treatment relative to the mean fold change of all peptides, highlighting peptides preferentially modulated by palbociclib.

Using these data, we performed GO term enrichment on the 127 peptides significantly enriched in low-dose-treated SKMEL5 cells (Fig. 4e), and identified enriched biological processes of interest, including ribosomal biogenesis, glucose metabolic process, and antigen processing, a reflection of the expected biological response to palbociclib^7,36,37^ (Fig. 4f, Supplementary Fig. 3f). We performed the same analysis with the raw, non-normalized values, and found only 66 of these peptides were significantly enriched without hipMHC quantification correction, altering the Gene Oncology (GO) term pathway analysis results (Supplementary Fig. 3g). While peptides mapping to ribosomal biogenesis were still significantly enriched, the other two biological processes were not, underscoring the importance of using hipMHCs for quantification correction to accurately interpret alterations in pMHC repertoires.

To determine if the measured pMHC alterations to SKMEL5 cells were common across cell lines, we compared the source proteins of peptides that were significantly enriched with low-dose treatment compared to DMSO across all four cell lines. Surprisingly, a majority (72–88%) of enriched source proteins were unique to each cell line, and we discovered only three proteins in common: vimentin, putative β-actin-like protein 3, and SIL1 nucleotide exchange factor (Fig. 4g, Supplementary Fig. 3h). Even when comparing source proteins of all peptides significantly increasing to any extent, just 17 proteins in common were identified, further illustrating the uniqueness of the proteins altered by palbociclib in each immunopeptidomic landscape (Supplementary Fig. 3i). We investigated whether the commonality of these 17 proteins could be explained by having high abundance in the peptide mixtures, but in SKMEL5 cells they were scattered throughout the distribution of AUC intensities (Supplementary Fig. 3j).

While the list of shared pMHCs and source proteins in common is limited, of interest is the serine-phosphorylated IRS2 (pIRS2) peptide, RVA[pS]PTSGVK. This post-translationally modified sequence has previously been shown to be restricted to malignant cells, with only the phosphorylated form demonstrating immunogenic potential^38,39^. Even though there are no alleles in common across the four cell lines^40^ (Supplementary Fig. 3k), we observed the pIRS2 peptide increasing across all cell lines with low-dose treatment (Fig. 4h). Furthermore, RVA[pS]PTSGVK has high expression among pMHCs (Supplementary Fig. 3j), and can be isolated without phospho-enrichment^41^. As a result, this peptide may be uniquely positioned as a broadly targetable antigen whose expression can be modulated by CDK4/6 inhibition. As a general effect of palbociclib treatment, TAAs derived from proteins like MLANA (MART1), PMEL (gp100), and TYR, among others, also increased in presentation following treatment (Fig. 4i). While these antigens and their source proteins are not universally conserved across our cell lines, the effect of increased TAA presentation following 1 μM palbociclib treatment could be applied to increase antigen presentation prior to immunotherapies targeting these well-documented antigens.

### Response to palbociclib is reflected in the immunopeptidome

**Fig. 5:**
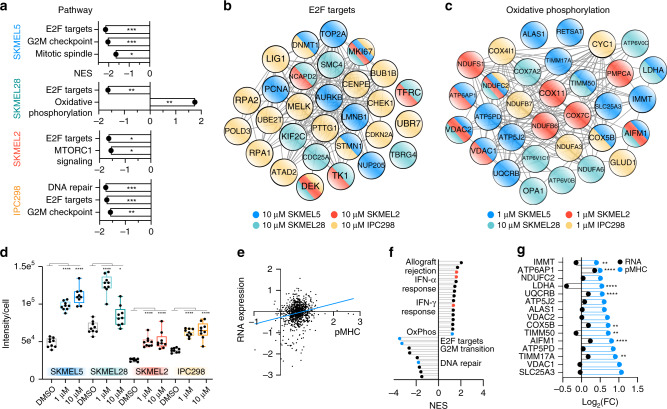
Pathway analysis of palbociclib-altered immunopeptidome. **a** Normalized enrichment score (NES) of significantly enriched pathways with 10 μM palbociclib, where +/− NES scores reflect enrichment directionality. For all, *q* < 0.25, and *p < 0.05, **p < 0.01, and ***p < 0.001. **b**, **c** String network of protein–protein interactions of all source proteins from E2F peptides (**b**) significantly decreasing with 10 μM palbociclib, and OxPhos peptides (**c**) significantly increasing with 1 μM palbociclib for all cell lines, except SKMEL28, where peptides from 10 μM are depicted. Node color corresponds to cell line. **d** Quantification (*n* = 9) of MitoTraker green intensity normalized to cell number following 72 h palbociclib treatment. Data are represented as a box and whiskers plot, with whiskers displaying minimum and maximum signal. Significance was determined using Dunnett’s multiple comparisons test for each condition versus DMSO. *p < 0.05 and ****p < 0.0001. **e** Correlation between log_2_ fold change (FC) of (palbociclib/DMSO) for RNA expression (*y*-axis) and pMHC presentation (*x*-axis) of SKMEL5 cells treated for 72 h with 1 μM palbociclib, *r*^2^ = 0.04. FC is calculated from the mean intensity of *n* = 3 biological replicates per condition. **f** Significantly enriched pathways using RNA-seq data (*p* < 0.05, *q* < 0.25). Annotated pathways reflect pathways also identified in the immunopeptidome analysis (blue), and those that match with previous reported data (red)^7^. **g** Log_2_(FC) for SKMEL5 OxPhos peptides significantly increasing (*p* < 0.05, blue) with 1 μM palbociclib, and matched log_2_(FC) of RNA expression (black). Significant differences in RNA expression (palbociclib versus DMSO) are indicated. **p < 0.01 and ****p < 0.0001 (Wald test, Benjamini–Hochberg (BH) adjusted). Exact significance values and other Source data are reported in the Source Data File.

To further assess whether quantitative differences in the immunopeptidome after palbociclib treatment are reflective of the cell signaling response to a perturbation, we performed a nonparametric test to identify positively and negatively enriched pathways. Gene names for source proteins were rank ordered according to fold change with treatment and searched against the MSigDB Hallmarks gene set database using Gene Set Enrichment Analysis (GSEA)^42–44^. This analysis did not reveal any significantly enriched pathways for the low-dose treatment, but high-dose palbociclib showed significant enrichment among downregulated pMHCs of E2F targets, G2M checkpoint, DNA repair, mitotic spindle, and MTORC1 signaling pathways in one or more cell lines (Fig. 5a). These findings reflect the known biological effects of CDK4/6 inhibition. For instance, inhibiting CDK4/6 decreases expression of E2F targets, and peptides derived from E2F targets like Ki-67, a proliferation marker, were depleted in all four cell lines (Fig. 5b). E2F also controls genes involved in DNA damage repair, and consistently, γH2AX levels, a marker of DNA double-strand breaks, increased at 72 h with palbociclib treatment in a dose-dependent manner^45^ (Supplementary Fig. 4a). Although similar biological processes are enriched across the four cell lines, source proteins for significantly depleted E2F peptides showed little overlap between the cell lines (Fig. 5b), again emphasizing the individuality of the source proteins contributing to each cell line’s detected pMHC repertoire.

Only one pathway, oxidative phosphorylation (OxPhos), was significantly upregulated in SKMEL28 cells. However, all cell lines presented peptides derived from the OxPhos pathway that increased significantly with palbociclib treatment, although this effect was more prominent with low-dose treatment in SKMEL5, IPC298, and SKMEL2 cells, in contrast to the results of SKMEL28 cells (Fig. 5c). OxPhos has been shown to increase with CDK4/6 inhibition due to increased ATP levels and mitochondrial mass, elevating metabolic activity. Comparably, all samples showed elevated mitochondrial levels following treatment, suggesting that enriched pMHC presentation of OxPhos-derived peptides reflects a change in the metabolic cell state (Fig. 5d).

Because alterations to the pMHC repertoire align with previously characterized biological responses to CDK4/6 inhibition, we tested whether changes in RNA expression could predict the quantitative immunopeptidome changes (Supplementary Data 4). No bulk correlation (*r*^2^ = 0.04) was observed between pMHC expression and RNA expression (Fig. 5e). This was unsurprising, as many mechanisms beyond gene expression regulate pMHC presentation, including protein synthesis, degradation, post-transitional modifications, processing, and more. Despite this poor correlation, significantly enriched gene sets in the immunopeptidome were also present in our RNA-sequencing (RNA-seq) analysis (Fig. 5f). While E2F pMHCs significantly depleted in SKMEL5 cells correlated with significantly decreased gene expression of the same source proteins (Supplementary Fig. 3b), only five of the 15 positively enriched OxPhos peptides displayed significantly higher gene expression with palbociclib treatment, with three decreasing in expression, and seven remaining unchanged (Fig. 5g). Collectively, these data suggest that while changes in gene expression and pMHC repertoires map to the same biological pathways, individual gene expression changes are not necessarily predictive of alterations in the immunopeptidome.

### IFN-γ-induced pMHC alterations are distinct from palbociclib

**Fig. 6:**
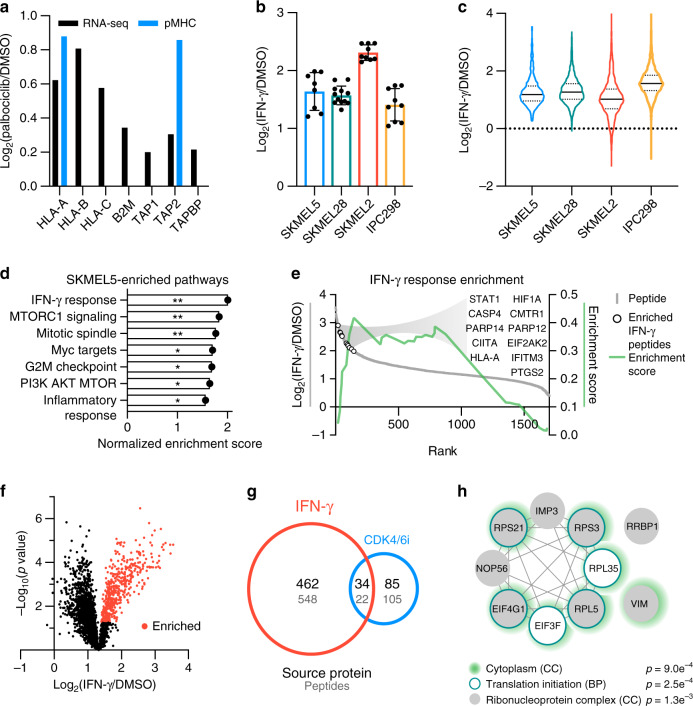
Quantifying the pMHC repertoire response to IFN-γ stimulation. **a** RNA-seq (black) and pMHC (blue) log_2_ fold change (FC) of 1 μM palbociclib/DMSO, calculated from the mean intensity of *n* = 3 biological replicates per condition, for antigen-processing genes. **b** Surface HLA expression via flow cytometry of cells treated with 72 h IFN-γ shown as log_2_(FC) (IFN-γ/DMSO). Errors bars represent ± SD, biological replicates are *n* = 8, 11, 9, and 9 for SKMEL5, SKMEL28, SKMEL2, and IPC298, respectively. **c** Immunopeptidome log_2_(FC), dotted lines display quartiles, and mean fold changes (solid line) are 2.42, 2.50, 2.08, and 3.04 for SKMEL5, SKMEL28, SKMEL2, and IPC298 cells, respectively. **d** Significantly enriched pathways in SKMEL5 cells with 72 h IFN-γ, *q* < 0.25, *p < 0.05, **p < 0.01. **e** Enrichment plot of IFN-γ response enrichment in SKMEL5 cells displays running enrichment score (green, right *y*-axis), and the log_2_(FC) (left *y*-axis) versus rank (*x*-axis) for each peptide (gray). Open circles show significantly enriched IFN-γ peptides. **f** Volcano plot of IFN-γ-induced changes in SKMEL5 cells. Peptides are presented as the log_2_(FC) versus mean-adjusted *p* value (unpaired two-sided *t* test). Red points represent peptides significantly enriched (*p* < 0.05, fold change > 2.42). **g** Venn diagram of significantly enriched source proteins (black) or peptides (gray) between IFN-γ and 1 μM palbociclib-treated SKMEL5 cells. **h** Protein–protein interaction network of significantly enriched source proteins in common, annotated by enriched gene ontology cellular components (CCs) and/or biological professes (BPs). Significance values are false discovery rate (FDR) adjusted. Exact significance values and other Source data are reported in the Source Data File.

Previous work has demonstrated that CDK4/6 inhibition stimulates IFN signaling, augmenting antigen presentation levels^15^. We also observed upregulation of IFN-γ response genes with low-dose palbociclib treatment, as well as increased expression of genes relating to antigen presentation (Figs. 5f,  6a). Consequently, we tested whether direct IFN-γ stimulation would shift the repertoire similarly to CDK4/6 inhibition. Cells were stimulated with DMSO or 10 ng mL^−1^ IFN-γ for 72 h and the resulting pMHC repertoires were quantified using our multiplexed hipMHC platform (Supplementary Data 5). IFN-γ increased surface pMHC levels >2× for each cell line (Fig. 6b), a trend that was reflected in the immunopeptidome, as nearly every identified pMHC increased in presentation with stimulation (Fig. 6c, Supplementary Fig. 5a).

To determine the similarity of response to palbociclib treatment, we again performed GSEA against the hallmark gene sets. The most significantly upregulated pathway in SKMEL5 cells with IFN-γ stimulation was the “IFN-γ response,” including peptides derived from proteins involved in antigen processing like STAT1 and HLA-A, in line with previous findings^46^ (Fig. 6d, e). In fact, IFN-γ response was the top enriched pathway in every cell line, reiterating that the cellular response to stimulus is reflected in quantitative differences in pMHC presentation, and that IFN-γ-related peptides are preferentially upregulated by IFN-γ stimulation (Supplementary Fig. 5b). Other pathways such as G2M checkpoint and mitotic spindle were positively enriched in IFN-γ stimulated cells, in contrast to the results of palbociclib treatment.

Although the cell lines showed differential pMHC pathway enrichment upon CDK4/6 inhibition with palbociclib and IFN-γ stimulation, we tested whether any pMHCs or source proteins were commonly enriched in response to these perturbations. In SKMEL5 cells, we identified just 20 peptides and 31 source proteins significantly enriched in both conditions (Fig. 6f, g), which primarily map to the cytoplasm and contain multiple ribosomal and translation initiation proteins frequently overrepresented in immunopeptidomic data sets (i.e., DRiPs)^47^ (Fig. 6h). These data demonstrate that while CDK4/6 inhibition may induce an IFN-γ response, stimulating cells with IFN-γ does not recapitulate the distinct peptide repertoire alterations observed with palbociclib treatment. Instead, IFN-γ stimulation alters the repertoire by augmenting the presentation of IFN-γ-related peptides.

## Discussion

The addition of hipMHCs as internal standards improves relative quantitative accuracy for both LF and multiplexed, labeled analyses, although multiplexed labeling with TMT showed superior accuracy and peptide binding specificity and yielded a higher number of quantifiable unique peptides using equivalent sample input. These internal hipMHC standards, which travel through the entire pMHC workflow, also account for variation across samples and provide an estimate for dynamic range suppression, which varies across peptides. We demonstrate that hipMHC correction alters the biological interpretation of quantitative pMHC repertoire changes, even in a relatively simple, in vitro system. Utilizing hipMHCs will be increasingly beneficial in accounting for variation in sample losses across heterogeneous in vivo samples, and in large studies to compare and correct quantitation across many multiplexed analyses or clinical sites. While we use TMT 6-plex in our analyses, this method is compatible with other isobaric labeling strategies, including iTRAQ (isobaric tags for relative and absolute quantitation), TMT 11-plex, and TMTpro, to analyze up to 16 samples simultaneously. For rapid profiling of immunopeptidome changes, we elected to use minimal sample input, making this protocol easily translatable for in vivo-derived tissue (e.g., clinical and animal) samples. While further reductions in sample input mirroring the amount obtained with a 14-gauge needle biopsy^48^ resulted in a notable decrease in the number of unique peptides identified (Supplementary Fig. 1f), we believe advancements in the speed and sensitivity of mass spectrometers, as well as in sample preparation techniques to reduce sample losses will enable pMHC profiling at even lower sample inputs in the future. Alternatively, using this same general platform of hipMHCs and isobaric multiplex labeling, the sample amount could be increased and coupled with fractionation for deeper sequencing of the pMHC repertoire, including neoantigen identification^49^.

In addition to improved relative quantification, we also demonstrated the utility of hipMHCs for pMHC absolute quantification by generating an embedded multipoint standard curve. Using targeted MS to detect attomole levels of antigen from just 1 × 10^7^ cells and regressing this signal against the titrated hipMHC standard, we were able to extract accurate absolute quantification in terms of copies per cell for two pMHC’s with ~20-fold difference in abundance. While absolute quantification is limited to just two peptides in this study, applying advanced targeted MS methods could enable the quantitation of hundreds of peptides in a single analysis^50^. The ability to readily determine the absolute quantification of detectable antigens of interest without the need for a pMHC-specific antibody will aid in targeted immunotherapy design. For instance, peptides of lower abundances may be better suited for engineered TCR-based therapies, as TCRs have been shown to be incredibly sensitive with as few as one pMHC complex being capable of initiating detectable T cell activation^51^. Alternatively, antibody-based therapies targeting specific pMHCs, for example, bi-specific T cell engagers or antibody–drug conjugates, may benefit from higher antigen expression levels, although results vary across antigen targets and antibody affinities^52^. Moreover, absolute quantification of pMHC expression can help to untangle the biological relationships among antigen processing, epitope abundances, immunogenicity, and off-target toxicity (e.g., tumor versus non-tumor abundance).

It is worth noting that one existing restriction to using hipMHCs is the commercial availability of UV-mediated MHC monomers and ELISA control reagents, which are limited to a handful of common human class I alleles. While matched allele hipMHCs are not required for normalization correction if MHC molecules are isolated using a pan-specific antibody, they are necessary for accurate absolute quantification with embedded standard curves. An analogous technology, disulfide-stabilized HLA molecules, could be used in place of UV-mediated exchange^53^. These HLA–B2M complexes show increased stability and higher exchange efficiency of lower-affinity peptides, potentially eliminating the need for an ELISA to quantify exchange efficiency and simplifying MHC refolding to expand this protocol to other alleles and species.

We applied our quantitative multiplexed hipMHC normalization to determine the pMHC repertoire response to CDK4/6 inhibition with palbociclib treatment in melanoma. These results indicate that extracellular changes in pMHC abundance are reflective of the intracellular response to CDK4/6 inhibition. Moreover, palbociclib treatment increased the presentation of TAAs and peptides derived from metabolic processes. Recently, high tumor antigen and metabolic protein expression levels have been shown to be predictive of checkpoint inhibitor response in melanoma, suggesting that palbociclib could be used in conjunction with CB- or TIL-based therapies to increase tumor immunogenicity^54^. As an alternate therapeutic strategy, peptide antigens whose surface expression was selectively increased by palbociclib could be utilized for targeted immunotherapy, either alone or in combination.

Indeed, the landscape of clinical trials exploring combination treatment regimens coupling checkpoint blockade with other therapies is rapidly expanding^55–57^. Quantifying the molecular consequences of these combination regimes with our platform could provide insight into these trials and enable the informed design of new therapeutic combinations, potentially with targeted immunotherapies. Taken together, our relative and absolute quantitative immunopeptidomic data demonstrate the utility of quantitative immunopeptidomics in evaluating the pMHC repertoire response to therapy. The multiplexed nature of this platform allows for analyses of many samples in a short timescale, an important feature in the context of clinical trials. Further analyses of pMHC repertoire changes will be useful in understanding the order and timing of therapies to achieve optimal success and may enable predictions as to how to tune the immunopeptidome to be most applicable to immunotherapy targeting.

## Methods

### Human cell lines

SKMEL5, SKMEL28, and MDA-MB-231 cell lines were obtained from ATCC (ATCC HTB-70, ATCC HTB-72, and HTB-26, respectively) and maintained in DMEM (Dulbecco’s modified Eagle’s medium) (Corning). IPC298 and SKMEL2 cells were provided by Array Biopharma and maintained in RPMI-1640 (Gibco) and minimum essential medium-α (Gibco), respectively. All media were supplemented with 10% fetal bovine serum (FBS) (Gibco) and 1% penicillin/streptomycin (Gibco). Cells were routinely tested for mycoplasma contamination, and maintained in 37 °C, 5% CO_2_.

### Phenotypic assays

IC_50_ of palbociclib (Selleckchem, PD-0332991) were determined for each cell line using CellTiter-Glo luminescent cell viability assay (Promega). Cells were seeded at density of 10,000 (SKMEL2, SKMEL28, IPC298) or 5,000 (SKMEL5) cells per well in a 96-well plate and allowed to adhere overnight. Cells were then treated with palbociclib or DMSO as a vehicle control in fresh medium for 72 h and assayed. Data were acquired using a Tecan plate reader Infinite 200 with Tecan icontrol version 1.7.1.12. IC_50_ values were calculated using a four-parameter logistic curve in Prism 8.4.1.

Mitochondrial content was measured using a fluorescent mitochondrial stain. Cells were seeded at a density of 20,000 cells per well in a 24-well plate and allowed to adhere overnight. Cells were then treated with 1 μM or 10 μM palbociclib or DMSO vehicle control in fresh medium for 72 h. Cells were assayed by incubating 200 nM of MitoTracker Green FM (Thermo Fisher) and a 1:1000 dilution of NuclearID Red DNA stain (Enzo Life Biosciences) for 15 min in a serum-free medium at 37 °C. After staining and medium exchange, cells were imaged and analyzed using the Incucyte Live Cell Analysis System (IncuCyte Zoom version 6.2.9200.0, Essen BioScience). The integrated intensity of MitoTracker dye was calculated for each image (*n* = 3 experimental replicates, *n* = 3 images per sample) and divided by the number of cells (counted using nuclear counterstain) to determine the mitochondrial intensity per cell. A one-way analysis of variance followed by Dunnett’s multiple comparisons statistical test was performed in Prism to compare the significance of treated cells versus vehicle DMSO control. Significance values represent multiplicity-adjusted *p* values.

### Flow cytometry

For analysis of cells by flow cytometry, cells were lifted with 0.05% Trypsin-EDTA and 10^6^ cells/mL were spun at 300 × *g* for 3 min, washed with ice-cold phosphate-buffered saline (PBS) supplemented with 1% FBS and 0.1% sodium azide (flow buffer), and incubated with fluorophore-conjugated antibody at 0.5 μg mL^−1^ in flow buffer for 30 min on ice. After incubation, cells were washed again, and resuspended in flow buffer plus 5 μL of propidium iodide staining solution (10 μg mL^−1^, Invitrogen) per sample. Analyses were performed on an LSRII (BD Biosciences) and data were analyzed using FlowJo (version 10.6.2). All antibodies were purchased from BioLegend: Alexa Fluor 488 HLA-A, -B, -C, clone W6/32 (cat. # 311413), Alexa Fluor 488 anti-H2A.X Phospho (Ser139), clone 2F3 (cat. # 613406). The gating strategy used for all experiments is provided in Supplementary Fig. 6.

### UV-mediated peptide exchange for hipMHCs

UV-mediated peptide exchange was performed using recombinant, biotinylated Flex-T HLA-A*02:01 monomers (BioLegend), using a modified version of the commercial protocol. Briefly, 4 μL of 500 μM peptide stock, 2 μL of Flex-T monomer, and 32 μL of 1× PBS were combined in a 96-well U-bottom plate. On ice, plates were illuminated with UV light (365 nm) for 30 min, followed by a 30-min incubation at 37 °C protected from light. Concentration of stable complexes following peptide exchange was quantified using the Flex-T HLA class I ELISA assay (BioLegend) as per the manufacturer’s instructions for HLA-A*02:01. ELISA results were acquired using a Tecan plate reader Infinite 200 with Tecan icontrol version 1.7.1.12.

### pMHC isolation

Cultured cells were seeded in 10 cm plates, allowed to adhere overnight, and treated for 72 h with palbociclib, 10 ng mL^−1^ human recombinant IFN-γ (ProSpec Bio), or DMSO vehicle control. At the time of harvest, cells were washed with 1× PBS, and lifted using 0.05% Trypsin-EDTA (Gibco). Cells were pelleted at 500 × *g* for 5 min, washed twice more in 1× PBS, and pelleted again. Cells were resuspended in 1 mL lysis buffer [20 nM Tris-HCl pH 8.0, 150 mM NaCl, 0.2 mM PMSO, 1% CHAPS, and 1× HALT Protease/Phosphatase Inhibitor Cocktail (Thermo Fisher)], followed by brief sonication (3 × 10 s microtip sonicator pulses) to disrupt cell membranes. The lysate was cleared by centrifugation at 5000 × *g* for 5 min and quantified using Bicinchoninic Acid Protein Assay Kit (Pierce).

pMHCs were isolated from 1 × 10^7^ cells per condition with IP and size-exclusion filtration, as previously described^58^ Briefly, for each condition 0.5 mg of pan-specific anti-human MHC class I (HLA-A, HLA-B, HLA-C) antibody [clone W6/32, Bio X Cell (cat. # BE0079)] was bound to 20 μL FastFlow Protein A Sepharose bead slurry (GE Healthcare) for 3 h rotating at 4 °C. Beads were washed 2× with IP buffer (20 nM Tris-HCl pH 8.0, 150 mM NaCl) prior to lysate and hipMHC addition, and incubated rotating overnight at 4 °C to isolate pMHCs. Beads were washed with 1× TBS and water, and pMHCs were eluted in 10% formic acid for 20 min at room temperature (RT). Peptides were isolated from antibody and MHC molecules using a passivated 10 K molecule weight cut-off filters (PALL Life Science), lyophilized, and stored at −80 °C prior to analysis.

### pMHC labeling with TMTs and SP3 cleanup

For labeled analyses, 100 μg of pre-aliquoted TMT 6-plex (TMT) was resuspended in 30 μL anhydrous acetonitrile (MeCN), and lyophilized peptides were resuspended in 100 μL 150 mM triethylammonium bicarbonate and 50% ethanol. Both were gently vortexed, centrifuged at 13,400 × *g* for 1 min, and combined. TMT/peptide mixtures were incubated on a shaker for 1 h at RT, followed by 15 min of vacuum centrifugation. After combining labeled samples, we washed tubes 2× with 25% MeCN in 0.1% acetic acid (AcOH) and added it to the labeled mixture, which was subsequently centrifuged to dryness.

Sample cleanup was performed using single-pot solid-phase-enhanced sample preparation (SP3) as previously described^59^. Briefly, a 1:1 mix of hydrophobic/hydrophilic Sera-mag carboxylate-modified speed beads (GE Healthcare) was prepared at a final bead concentration of 10 μg μL^−1^. Labeled samples were resuspended in 30 μL of 100 mM ammonium bicarbonate (pH 7–8) and added to 500 μg of bead mix with 1 mL MeCN. Peptides were allowed to bind for 10 min at RT, washed 2× with MeCN, and eluted with 2% DMSO for 1 min of sonication in a bath sonicator. TMT-labeled peptides were transferred to a fresh microcentrifuge tube and centrifuged to dryness.

### Synthetic peptide standards

Heavy leucine-containing peptides were synthesized at the MIT Biopolymers and Proteomics Lab using standard Fmoc chemistry using an Intavis model MultiPep peptide synthesizer with HATU activation and 5 μmol chemistry cycles. Starting resin used was Fmoc-Amide Resin (Applied Biosystems). Cleavage from resin and simultaneous amino acid side chain deprotection was accomplished using: trifluoroacetic acid (81.5% v/v); phenol (5% v/v); water (5% v/v); thioanisole (5% v/v); 1,2-ethanedithiol (2.5% v/v); 1% triisopropylsilane for 1.5 h. Standard Fmoc amino acids were procured from NovaBiochem and Fmoc-Leu (^13^C_6_, ^15^N) was obtained from Cambridge Isotope Laboratories.

Peptides were quality controlled by MSy and reverse phase chromatography using a Bruker MiroFlex MALDI-TOF and Agilent model 1100 HPLC system with a Vydac C18 column (300 Å, 5 μm, 2.1 × 150 mm^2^) at 300 μL/min monitoring at 210 and 280 nm with a trifluoroacetic acid/H_2_O/MeCN mobile phase survey gradient. All peptides contain C-terminal amidation, with the exception of the BCAP31 and DDX5 peptides used for absolute quantification. For amidated peptides, we observe C-terminal amidation and C-terminal carboxyl groups on peptides synthesized with an amide group. Therefore, both are considered in downstream analyses.

### RNA-sequencing

RNA was isolated from 10 cm plates of SKMEL5 cells with three biological replicates per condition. Prior to harvest, cells were washed with ice-cold 1× PBS over ice and lysed in TRIzol reagent (Thermo Fisher). Total RNA was isolated from each sample using Direct-zol RNA MiniPrep kit (Zymo Research) according to the manufacturer’s instructions.

RNAs were confirmed for quality using the Agilent Fragment Analyzer and 300 ng of material was polyA selected using NEBNext Poly(A) mRNA Magnetic Isolation Module (E7490) modified to include two rounds of polyA binding and 10 min incubations. cDNA was generated using the NEB Ultra II Directional Kit (E7760) following the manufacturer’s protocol using 12 cycles of PCR and a 0.9X SPRI clean. The resulting libraries were quality assessed using the Fragment Analyzer and quantified by quantitative PCR prior to be sequenced on the Illumina HiSeq2000. The 40 nt single-end reads with an average depth of five million reads per sample were sequenced for all conditions.

RNA-seq reads were aligned to the human transcriptome prepared with the hg38 primary assembly and the Ensembl version 95 annotation using STAR version 2.5.3a^60^. Gene expression was summarized with RSEM version 1.3.0 and SAMtools version 1.3 (refs^61,62^). Differential expression analysis was performed with DESeq2 version 1.24.0 running under R version 3.6.0 with normal log fold change shrinkage^63^. Significance values (adjusted *p* value) are determined using the Wald test, and are multiple hypothesis corrected using Benjamini–Hochberg method. The resulting data were parsed and assembled using Tibco Spotfire Analyst version 7.11.1.

### MS data acquisition

For MS analysis, peptides were resuspended in 0.1% AcOH and loaded on a precolumn packed in-house [100 μm ID × 10 cm packed with 10 μm C18 beads (YMC gel, ODS-A, 12 nm, S-10 μm, AA12S11)]. The precolumn was then washed with 0.1% AcOH and connected in series to an analytical capillary column with an integrated electrospray tip (~1 μm orifice) with 5 μM C18 beads, prepared in-house [(50 μm ID × 12 cm with 5 μm C18 beads (YMC gel, ODS-AQ, 12 nm, S-5 μm, AQ12S05)].

Peptides were eluted using a 130-min gradient with 10–45% buffer B (70% MeCN, 0.2 M AcOH) from 5 to 100 min and 45–55% buffer B from 100 to 120 min at a flow rate of 0.2 mL/min for a flow split of ~10,000:1. Peptides were analyzed using a Thermo Fisher Q Exactive HF-X Hybrid Quadrupole-Orbitrap mass spectrometer, and data were acquired using Thermo Fisher Scientific Xcalibur version 2.9.0.2923. Standard MS parameters were as follows: spray voltage, 2.5 kV; no sheath or auxiliary gas flow; heated capillary temperature, 250 °C.

The HF-X was operated in DDA mode for LF and TMT analyses. LF: Full-scan MS spectra (mass/charge ratio (*m/z*), 350–2000; resolution, 60,000) were detected in the Orbitrap analyzer after accumulation of ions at 3e^6^ target value with a maximum injection time (IT) of 50 ms. For every full scan, the top 20 most intense ions were isolated (isolation width of 0.4 *m*/*z*) and fragmented (collision energy: 28%) by higher energy collisional dissociation with a maximum injection time of 300 ms, automatic gain control target 1e^5^, and 60,000 resolution. Charge states <2 and >4 were excluded, and dynamic exclusion was set to 30 s. TMT: Full-scan MS spectra (*m/z*, 400–2000; resolution, 120,000) were detected in the Orbitrap analyzer after accumulation of ions at 3e^6^ target value with a maximum IT of 50 ms. For every full scan, the 20 most intense ions were isolated (isolation width of 0.4 *m*/*z*) and fragmented (collision energy: 29%) by higher energy collisional dissociation with a maximum injection time of 350 ms, AGC target 1e^5^, and 30,000 resolution. Charge states <2 and >4 were excluded, and dynamic exclusion was set to 60 s. To ensure fragmentation of normalization standards, one fraction may be analyzed using targeted selected ion monitoring used in tandem with DDA with an inclusion list of hipMHC standards. For absolute quantification, the HF-X was operated in DDA mode with inclusion list enabled. Parameters mirror those of the TMT DDA method, with several exceptions. Full-scan mass spectra *m*/*z* range: 300–1200, maximum MS2 injection time 200 ms, only charge states of 2 and 3 were considered. Inclusion list masses and charge states listed in Supplementary Data 6.

### MS search space and filtering

All mass spectra were analyzed with Proteome Discoverer (PD, version 2.2) and searched using Mascot (version 2.4) against the human SwissProt database. No enzyme was used, and variable modifications included oxidized methionine for all analyses and phosphorylated serine, threonine, and tyrosine for cell treatment analyses. Treatment analyses were also searched against a previously published catalog of over 40,000 predicted antigenic mutations in cancer cell lines^64^. Heavy leucine-containing peptides were searched for separately with heavy leucine (+7), C-terminal amidation, and methionine oxidation as dynamic modifications against a custom database of the synthetic peptide standards. All analyses were filtered with the following criteria: search engine rank = 1, isolation interference ≤ 30%, and length between 8 and 15 amino acids. LF analyses were filtered with ion score ≥ 20, and labeled samples were filtered with ion score ≥15 and percolator *q* value ≤ 0.05. AUC quantitation was performed using the minora feature detector in PD with match between runs enabled and filtered for ion score ≥20. For targeted, absolute quantification analyses, total ion count values for each scan and peak intensities were extracted using Skyline (version 19.1.0.193)^65^.

### MS data analysis with hipMHC correction

For LF analyses, correction parameters were determined by calculating the ratio of AUC intensities in each sample against a reference sample and taking the mean across hipMHCs. For TMT-labeled samples, ratios against a reference channel (usually TMT126) were calculated and the median of all ratios for correction hipMHCs was used to determine the final correction parameters. Only PSMs of heavy leucine-coded peptides with an average reporter ion intensity within 10-fold of the interquartile range of endogenous PSM reporter ion intensities were used for correction, as we observed drift in the correction factors when PSM TMT intensities were well beyond endogenous levels. For absolute quantification analyses, correction factors were generated as described for TMT analyses, and used to normalize maximum peak intensity values for DDX5 and BCAP31. Notably, with mean fold changes >2× between samples (e.g., IFN-γ stimulation), in our hands hipMHCs are no longer able to correct between conditions despite narrow isolation window (0.4 *m*/*z*). This inaccuracy may be due to co-isolation, as the calculated correction factors reflect median fold changes of endogenous peptides. In this case, we generated correction factors for each treatment condition separately.

Correction factors were applied to AUC values in LF analyses for all peptides that were quantifiable across samples. For labeled samples, ion intensities of PSMs for each unique peptide across analyses of the same sample were summed, after which normalization factors were applied. To evaluate differences between conditions, the log_2_-transformed ratio of arithmetic mean intensity for drug- and DMSO-treated samples (*n* = 3) was calculated. To determine if peptides were significantly increasing, an unpaired, two-sided *t* test was performed, and peptides with *p* ≤ 0.05 were considered significantly increasing. To evaluate which peptides were significantly enriched above the mean, treated samples were mean centered by dividing the ion intensity of each peptide by the mean fold change across all peptides, after which a Student’s two-tailed *t* test was performed on adjusted values. Peptides with a mean-adjusted *p* value ≤ 0.05 were considered significantly enriched. Mean centering was not performed on samples where the mean log_2_ fold change was between −0.07 and 0.07. Data analyses were performed using Matlab version R2019b, and Microsoft Excel version 16.34.

### pMHC binding affinity

Binding affinity of pMHCs was estimated using NetMHCpan-4.0 against each cell line’s allelic profile^28,40^ (Supplementary Fig. 3k). Only 9-mers were evaluated, and the minimum predicted affinity (nM) of each peptide was used to assign peptides to their best predicted allele. The threshold for binding was set to 500 nM. Binding motifs for the alleles were generated using 9-mers with predicted affinity <500 nM, and visualized using WebLogo 2.8.2 (ref.^66^). To estimate the proportion of peptides predicted to be binders by chance, 10 sets of 2000 random 9-mers were created by selecting with equal probability any amino acid more than 8 amino acid from a protein C terminus as a start site from human proteins in SwissProt version 2019_2, and binding affinity prediction was performed against the alleles of MDA-MB-231 cells. Data presented in Fig. 2d are a representative example.

### Enrichment analyses

For pMHC pathway enrichment analyses, gene names from peptide source proteins were extracted and rank ordered according to the average log_2_ fold change over DMSO-treated cells. In cases where more than one peptide mapped to the same source protein, the maximum/minimum was chosen, depending on the directionality of enrichment analysis. For RNA-seq data, gene sets were rank ordered according to the mean log_2_ fold change value with only protein encoding genes considered. We utilized GSEA 4.0.3 pre-ranked tool against the Molecular Signatures Database hallmarks gene sets with 1000 permutations, weighted enrichment statistic (*p* = 1), and a minimum gene size of 8 for pMHC analyses and 15 for RNA-seq^42–44^. Results were filtered for false discovery rate *q* value ≤0.25, and nominal *p* value ≤ 0.05.

Significantly enriched peptides (mean-adjusted *p* value ≤0.05) were analyzed using STRING v.11 for GO term enrichment against biological processes and cellular components data sets^67,68^. Enriched categories were filtered according to false discovery rate *q* value ≤0.05.

### Reporting summary

Further information on research design is available in the Nature Research Reporting Summary linked to this article.

## Supplementary information

Supplementary Information

Description of Additional Supplementary Files

Supplementary Data 1

Supplementary Data 2

Supplementary Data 3

Supplementary Data 4

Supplementary Data 5

Supplementary Data 6

Reporting Summary

## Data Availability

RNA-sequencing data have been deposited into the NCBI Gene Expression Omnibus GSE144373. The mass spectrometry proteomics data have been deposited to the ProteomeXchange Consortium via the PRIDE partner repository with the data set identifier PXD017407. Analyzed mass spectrometry immunopeptidomics data from analyses in Figs. 2–6 is available in Supplementary Data 1, 2, 3, and 4, respectively. File maps linking files to corresponding data sets in the manuscript is available in Supplementary Data 1–3, 5. Analyzed RNA-seq data is available in Supplementary Data 3. The list of targeted masses used in Fig. 3 for absolute quantification of peptide MHCs is listed in Supplementary Data 6. The source data underlining Figs. 2d–h, 3a, c, d, 4a, d, e, h, 5a, d–g, 6b–d and Supplementary Figs. 1g, 3b, d, e, j, and 5a, b are provided as a Source Data File. All other data are available from the corresponding author on reasonable request.

## Source data

Source Data

## References

[1] Caron E (2011). The MHC I immunopeptidome conveys to the cell surface an integrative view of cellular regulation. Mol. Syst. Biol..

[2] Sharma P, Hu-Lieskovan S, Wargo JA, Ribas A (2017). Primary, adaptive, and acquired resistance to cancer iImmunotherapy. Cell.

[3] Martins F (2019). Adverse effects of immune-checkpoint inhibitors: epidemiology, management and surveillance. Nat. Rev. Clin. Oncol..

[4] Reits EA (2006). Radiation modulates the peptide repertoire, enhances MHC class I expression, and induces successful antitumor immunotherapy. J. Exp. Med..

[5] Brea EJ (2016). Kinase regulation of human MHC class I molecule expression on cancer cells. Cancer Immunol. Res..

[6] Liu WM, Fowler DW, Smith P, Dalgleish AG (2010). Pre-treatment with chemotherapy can enhance the antigenicity and immunogenicity of tumours by promoting adaptive immune responses. Br. J. Cancer.

[7] Goel S (2017). CDK4/6 inhibition triggers anti-tumour immunity. Nature.

[8] Sullivan RJ (2019). Atezolizumab plus cobimetinib and vemurafenib in BRAF-mutated melanoma patients. Nat. Med..

[9] Ascierto PA (2019). Dabrafenib, trametinib and pembrolizumab or placebo in BRAF-mutant melanoma. Nat. Med..

[10] Hunt D (1992). Characterization of peptides bound to the class I MHC molecule HLA-A2.1 by mass spectrometry. Science.

[11] Bassani-Sternberg M, Pletscher-Frankild S, Jensen LJ, Mann M (2015). Mass spectrometry of human leukocyte antigen class I peptidomes reveals strong effects of protein abundance and turnover on antigen presentation. Mol. Cell. Proteom..

[12] Khodadoust MS (2017). Antigen presentation profiling reveals recognition of lymphoma immunoglobulin neoantigens. Nature.

[13] Bogunovic B, Srinivasan P, Ueda Y, Tomita Y, Maric M (2010). Comparative quantitative mass spectrometry analysis of MHC class II-associated peptides reveals a role of GILT in formation of self-peptide repertoire. PLoS ONE.

[14] Shetty V (2012). Quantitative immunoproteomics analysis reveals novel MHC class I presented peptides in cisplatin-resistant ovarian cancer cells. J. Proteom..

[15] Jensen, S. M., Potts, G. K., Ready, D. B. & Patterson, M. J. Specific MHC-I peptides are induced using PROTACs. *Front. Immunol*. **9**, 2697 (2018). 10.3389/fimmu.2018.02697 PMC6262898 30524438

[16] Loffler MW (2018). Mapping the HLA ligandome of colorectal cancer reveals an imprint of malignant cell transformation. Cancer Res..

[17] Murphy JP (2019). Multiplexed relative quantitation with isobaric tagging mass spectrometry reveals class I major histocompatibility complex ligand dynamics in response to doxorubicin. Anal. Chem..

[18] Schittenhelm RB, Sian TCCLK, Wilmann PG, Dudek NL, Purcell AW (2015). Revisiting the Arthritogenic Peptide Theory: quantitative not qualitative changes in the peptide repertoire of HLA-B27 allotypes. Arthritis Rheumatol..

[19] Hassan C (2014). Accurate quantitation of MHC-bound peptides by application of isotopically labeled peptide MHC complexes. J. Proteom..

[20] Wang Q (2019). Direct detection and quantification of neoantigens. Cancer Immunol. Res..

[21] Hogan KT (2005). Use of selected reaction monitoring mass spectrometry for the detection of specific MHC class I peptide antigens on A3 supertype family members. Cancer Immunol. Immunother..

[22] Tan CT, Croft NP, Dudek NL, Williamson NA, Purcell AW (2011). Direct quantitation of MHC-bound peptide epitopes by selected reaction monitoring. Proteomics.

[23] Wu, T. et al. Quantification of epitope abundance reveals the effect of direct and cross-presentation on influenza CTL responses. *Nat. Commun*. **10**, 2846 (2019). 10.1038/s41467-019-10661-8 PMC6599079 31253788

[24] Bozzacco L (2011). Mass spectrometry analysis and quantitation of peptides presented on the MHC II molecules of mouse spleen dendritic cells. J. Proteome Res..

[25] Bijen Helena M., Hassan Chopie, Kester Michel G. D., Janssen George M. C., Hombrink Pleun, de Ru Arnoud H., Drijfhout Jan Wouter, Meiring Hugo D., de Jong Ad P., Falkenburg J. H. Frederik, Jimenez Connie R., Heemskerk Mirjam H. M., van Veelen Peter A. (2018). Specific T Cell Responses against Minor Histocompatibility Antigens Cannot Generally Be Explained by Absence of Their Allelic Counterparts on the Cell Surface. PROTEOMICS.

[26] Yang Y, Xiang Z, Ertl HCJ, Wilson JM (1995). Upregulation of class I major histocompatibility complex antigens by interferon γ is necessary for T-cell-mediated elimination of recombinant adenovirus-infected hepatocytes in vivo. Proc. Natl Acad. Sci. USA.

[27] Rodenko B (2006). Generation of peptide-MHC class I complexes through UV-mediated ligand exchange. Nat. Protoc..

[28] Jurtz V (2017). NetMHCpan-4.0: improved peptide–MHC class I interaction predictions integrating eluted ligand and peptide binding affinity data. J. Immunol..

[29] Bassani-Sternberg Michal, Chong Chloé, Guillaume Philippe, Solleder Marthe, Pak HuiSong, Gannon Philippe O., Kandalaft Lana E., Coukos George, Gfeller David (2017). Deciphering HLA-I motifs across HLA peptidomes improves neo-antigen predictions and identifies allostery regulating HLA specificity. PLOS Computational Biology.

[30] Gloger A, Ritz D, Fugmann T, Neri D (2016). Mass spectrometric analysis of the HLA class I peptidome of melanoma cell lines as a promising tool for the identification of putative tumor-associated HLA epitopes Europe PMC Funders Group. Cancer Immunol. Immunother..

[31] Nyamao RM, Wu J, Yu L, Xiao X, Zhang FM (2019). Roles of DDX5 in the tumorigenesis, proliferation, differentiation, metastasis and pathway regulation of human malignancies. Biochim. Biophys. Acta.

[32] Choi YJ, Anders L (2014). Signaling through cyclin D-dependent kinases. Oncogene.

[33] Hamilton E, Infante JR (2016). Targeting CDK4/6 in patients with cancer. Cancer Treat. Rev..

[34] Schettini F (2018). CDK 4/6 inhibitors as single agent in advanced solid tumors. Front. Oncol..

[35] Deng J (2018). CDK4/6 inhibition augments antitumor immunity by enhancing T-cell activation. Cancer Discov..

[36] Donati G, Montanaro L, Derenzini M (2012). Ribosome biogenesis and control of cell proliferation: p53 is not alone. Cancer Res..

[37] Franco J, Balaji U, Freinkman E, Witkiewicz AK, Knudsen ES (2016). Metabolic reprogramming of pancreatic cancer mediated by CDK4/6 inhibition elicits unique vulnerabilities. Cell Rep..

[38] Zarling AL (2006). Identification of class I MHC-associated phosphopeptides as targets for cancer immunotherapy. Proc. Natl Acad. Sci. USA.

[39] Zarling AL (2014). MHC-restricted phosphopeptides from insulin receptor substrate-2 and CDC25b offer broad-based immunotherapeutic agents for cancer. Cancer Res..

[40] Scholtalbers J (2015). TCLP: an online cancer cell line catalogue integrating HLA type, predicted neo-epitopes, virus and gene expression. Genome Med..

[41] Bassani-Sternberg M (2016). Direct identification of clinically relevant neoepitopes presented on native human melanoma tissue by mass spectrometry. Nat. Commun..

[42] Mootha VK (2003). PGC-1α-responsive genes involved in oxidative phosphorylation are coordinately downregulated in human diabetes. Nat. Genet..

[43] Subramanian A (2005). Gene set enrichment analysis: a knowledge-based approach for interpreting genome-wide expression profiles. Proc. Natl Acad. Sci. USA.

[44] Liberzon A (2015). The Molecular Signatures Database hallmark gene set collection. Cell Syst..

[45] Hashizume R (2016). Inhibition of DNA damage repair by the CDK4/6 inhibitor palbociclib delays irradiated intracranial atypical teratoid rhabdoid tumor and glioblastoma xenograft regrowth. Neuro Oncol..

[46] Chong C (2018). High-throughput and sensitive immunopeptidomics platform reveals profound interferon γ-mediated remodeling of the human leukocyte antigen (HLA) ligandome. Mol. Cell. Proteom..

[47] Bourdetsky, D., Schmelzer, C. E. H. & Admon, A. The nature and extent of contributions by defective ribosome products to the HLA peptidome. *Proc. Natl. Acad. Sci. USA* **111**, E1591–E1599 (2014). 10.1073/pnas.1321902111 PMC4000780 24715725

[48] Labots M (2017). Phosphotyrosine-based-phosphoproteomics scaled-down to biopsy level for analysis of individual tumor biology and treatment selection. J. Proteom..

[49] Purcell AW, Ramarathinam SH, Ternette N (2019). Mass spectrometry-based identification of MHC-bound peptides for immunopeptidomics. Nat. Protoc..

[50] Gallien S, Yoon Kim S, Domon B (2015). Large-scale targeted proteomics using internal standard triggered-parallel reaction monitoring (IS-PRM). Mol. Cell. Proteom..

[51] Huang J (2013). A single peptide-major histocompatibility complex ligand triggers digital cytokine secretion in CD4 + T Cells. Immunity.

[52] Ellerman D (2019). Bispecific T-cell engagers: towards understanding variables influencing the in vitro potency and tumor selectivity and their modulation to enhance their efficacy and safety. Methods.

[53] Moritz Andreas, Anjanappa Raghavendra, Wagner Claudia, Bunk Sebastian, Hofmann Martin, Pszolla Gabriele, Saikia Ankur, Garcia-Alai Maria, Meijers Rob, Rammensee Hans-Georg, Springer Sebastian, Maurer Dominik (2019). High-throughput peptide-MHC complex generation and kinetic screenings of TCRs with peptide-receptive HLA-A*02:01 molecules. Science Immunology.

[54] Harel M (2019). Proteomics of melanoma response to immunotherapy reveals mitochondrial dependence. Cell.

[55] Esteva FJ, Hubbard-Lucey VM, Tang J, Pusztai L (2019). Immunotherapy and targeted therapy combinations in metastatic breast cancer. Lancet Oncol..

[56] Yu, C. et al. Combination of immunotherapy with targeted therapy: theory and practice in metastatic melanoma. *Front. Immunol.* **10**, 990 (2019). 10.3389/fimmu.2019.00990 PMC6513976 31134073

[57] McGranahan, T., Therkelsen, K. E., Ahmad, S. & Nagpal, S. Current state of immunotherapy for treatment of glioblastoma. *Curr. Treat. Opt. Oncol.* **20**, 24 (2019). 10.1007/s11864-019-0619-4 PMC6394457 30790064

[58] Jaeger AM (2019). Rebalancing protein homeostasis enhances tumor antigen presentation. Clin. Cancer Res..

[59] Browne CM (2019). A chemoproteomic strategy for direct and proteome-wide covalent inhibitor target-site identification. J. Am. Chem. Soc..

[60] Dobin A (2013). STAR: Ultrafast universal RNA-seq aligner. Bioinformatics.

[61] Li H (2009). The Sequence Alignment/Map format and SAMtools. Bioinformatics.

[62] Li B, Dewey CN (2011). RSEM: accurate transcript quantification from RNA-Seq data with or without a reference genome. BMC Bioinform..

[63] Anders S, Huber W (2010). Differential expression analysis for sequence count data. Genome Biol..

[64] Boegel, S., Löwer, M., Bukur, T., Sahin, U. & Castle, J. C. A catalog of HLA type, HLA expression, and neoepitope candidates in human cancer cell lines. *Oncoimmunology* **3**, e954893 (2014). 10.4161/21624011.2014.954893 PMC4355981 25960936

[65] MacLean B (2010). Skyline: an open source document editor for creating and analyzing targeted proteomics experiments. Bioinformatics.

[66] Crooks GE, Hon G, Chandonia JM, Brenner SE (2004). WebLogo: a sequence logo generator. Genome Res..

[67] Szklarczyk D (2019). STRING v11: protein–protein association networks with increased coverage, supporting functional discovery in genome-wide experimental datasets. Nucleic Acids Res..

[68] Ashburner M (2000). Gene ontology: tool for the unification of biology. Nat. Genet..

