# Supplementary material for: Multiplexed relative and absolute quantitative immunopeptidomics reveals MHC I repertoire alterations induced by CDK4/6 inhibition
Source: Nat Commun. 2020 Jun 2;11:2760. doi: 10.1038/s41467-020-16588-9 (PMC7265461; doi:10.1038/s41467-020-16588-9)
Supplement: Supplementary file 1 — Supplementary Information [file 41467_2020_16588_MOESM1_ESM.pdf]

Supplementary Information

Multiplexed relative and absolute quantitative immunopeptidomics reveals MHC I repertoire alterations induced by CDK4/6 inhibition

Stopfer et al.

Supplementary Figure 1

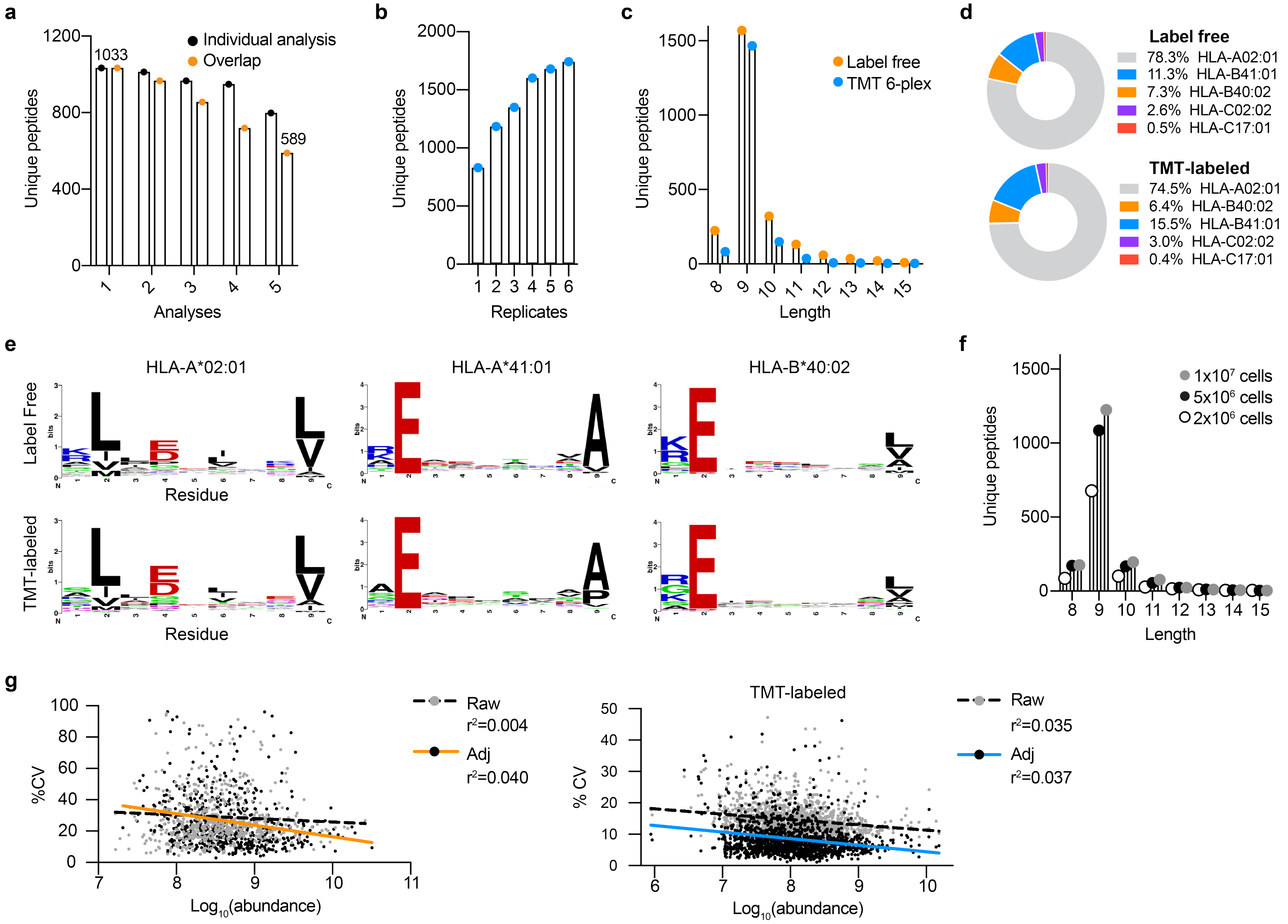

Supplementary Figure 1. a. LF analyses (n=5 technical replicates) of MDA-MB-231 cells show poor overlap of quantifiable peptides observed across multiple analyses (orange) compared to the number of peptides identified in any given single analysis (black). **b.** Total peptides identified combining the results from n=1 to n=6 independent analyses of TMT-labeled MDA-MB-231 cells, analyzing 15-20% of the peptide elution per analysis. **c.** Length distribution of MDA-MB-231 peptides for LF and TMT-labeled samples. Unique peptides are identified from the union of n=5 LF technical replicates and n=6 TMT-labeled elution fractions. **d.** Distribution of 9-mer peptides across HLA alleles with a predicted affinity < 500 nM for LF (top) and TMT-labeled (bottom) analyses. **e.** Binding motifs of alleles containing ≥ 5% of predicted binders for LF (top) and TMT-labeled (bottom) analyses. **f.** Number of unique peptides identified using different amounts of MDA-MB-231 cells as sample input. Values represent a single analysis using 20% of the peptide elution. **g.** Correlation of each peptide's coefficient of variation (CV) versus abundance for LF (left) and TMT-labeled (right) analyses.

Supplementary Figure 2

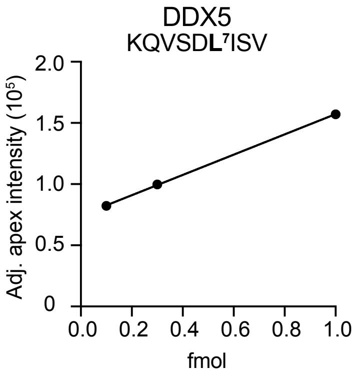

Supplementary Figure 2. Calibration curve of the DDX5 peptide (KLDVGNAEV) added into 1x10^7 MDA-MB -231 cells from 0.1 fmol to 1 fmol. 0.1 fmol corresponds to ~6 copies per cell.

Supplementary Figure 3

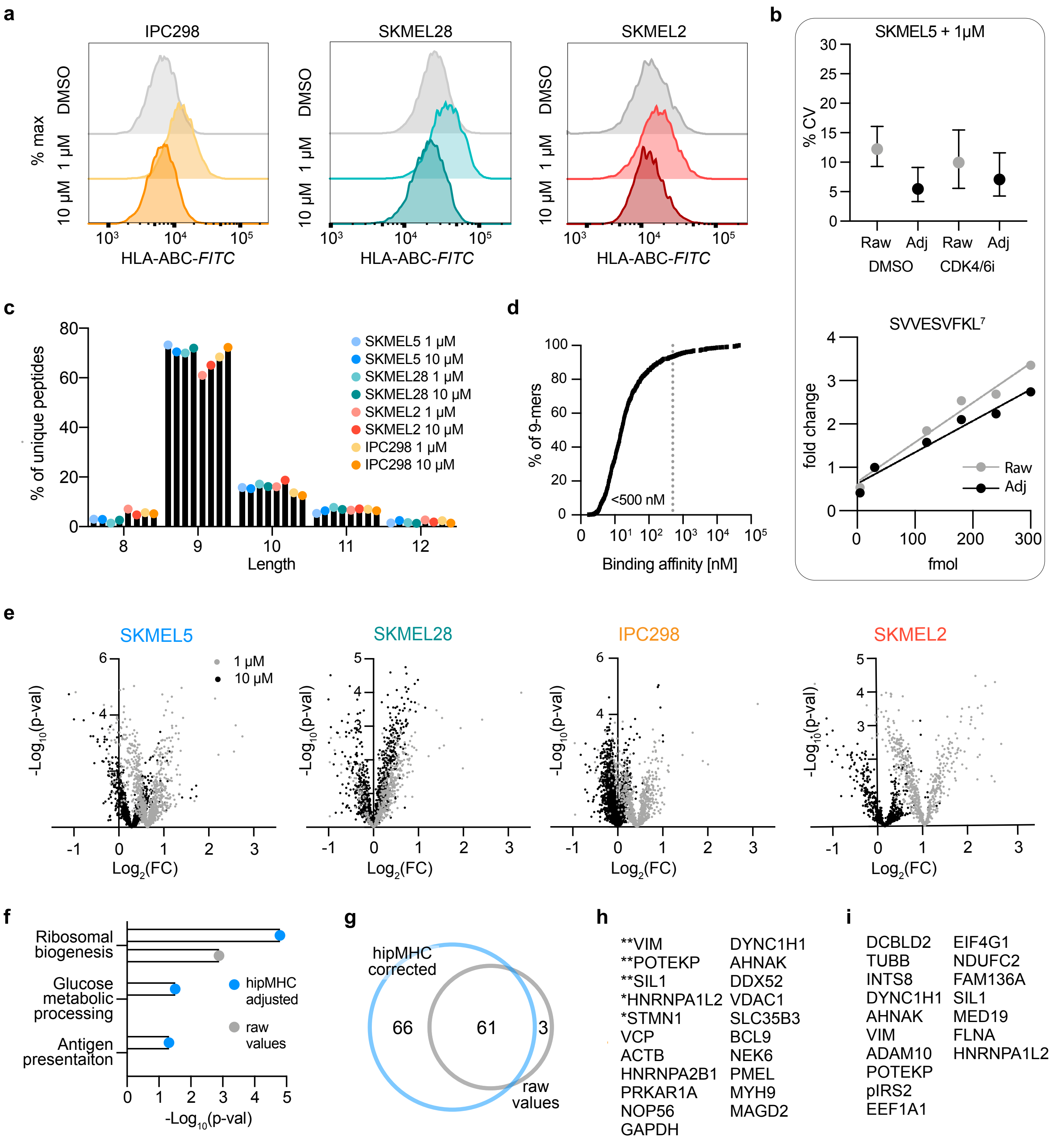

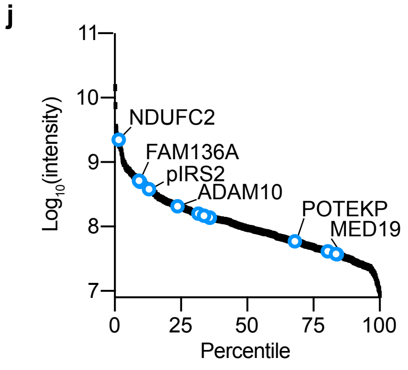

k

	HLA-A	HLA-B	HLA-C
SKMEL5	02:01 11:01	40:01 07:02	03:04 07:02
SKMEL28	11:01	40:01	03:04
IPC298	24:02 03:01	07:02 35:02	04:01 07:02
SKMEL2	26:01 03:01	38:01 35:08	04:01 12:03

Supplementary Figure 3

Supplementary Figure 3. a. Surface HLA expression with DMSO or palbociclib treatment for 72h measured via flow cytometry, presented as % of maximum signal. A representative plot of distributions observed among three replicates is shown. **b.** Applying hipMHC correction factors decreases the coefficient of variation in both DMSO and 1 μM palbociclib SKMEL5 cells (upper), where data are presented as mean values +/- SD for n=3 biological replicates. The titrated peptide, SVVESVFKL, displays 3.6x dynamic range suppression (lower). **c.** Length distribution of peptides identified in each cell line and treatment. Data is represented as % of total peptides identified. **d.** Predicted binding affinity of 9-mer peptides in SKMEL5 cells with DMSO or 1 μM palbociclib. 93.3% have a predicted affinity of <500 nM. **e.** Volcano plots representing $\log_2$ fold change (FC) of pMHC presentation of (palbociclib/DMSO) versus significance (mean adjusted p-value, unpaired two-sided t test). Data points are colored by treatment, 1 μM (grey) and 10 μM (black). **f.** Significantly enriched biological processes (GO term enrichment, FDR-adjusted $p < 0.05$) in SKMEL5 cells + 1 μM palbociclib using significantly enriched peptides from the raw data (grey) and hipMHC adjusted data (blue). **g.** Number of significantly enriched peptides in raw data (grey) versus hipMHC adjusted data (blue) for SKMEL5 cells +/- 1 μM Palbociclib. **h.** Source proteins of peptides significantly enriched (mean adjusted p-value) following palbociclib treatment, **= significantly upregulated in four cell lines, *= three lines, all others were seen in at least two cell lines. **i.** 17 common source proteins significantly increasing in all four cell lines. **j.** Average peptide AUC integrated abundance vs. percentile rank of abundance of all SKMEL5 +/- 1 μM palbociclib peptides. Blue points are peptides labeled by their source protein that are significantly increased in all four cell lines. **k.** Class I allelic profiles of each cell line.

Supplementary Figure 4

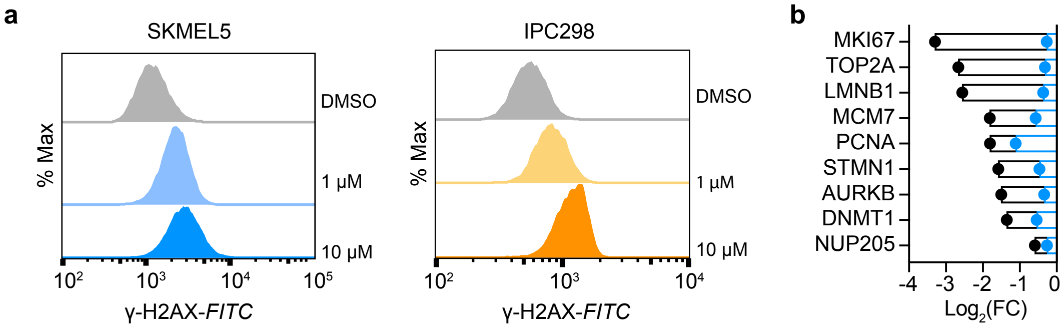

Supplementary Figure 4. **a.** Histogram of γ-H2AX levels determined by flow cytometry in cells treated with DMSO or 1 µM / 10 µM palbociclib for 72h. Data is represented as % of maximum signal, and results are representative of those obtained in a second independent experiment. **b.** Log_2 fold change (FC) for SKMEL5 E2F peptides significantly decreasing in presentation (blue) with 10 µM palbociclib for 72h and matched RNA expression of SKMEL5 cells treated with 1 µM palbociclib for 72h (black). Immunopeptidomics and RNA-sequencing experiments each contained n=3 biological replicates for each condition. RNA sequencing was not performed on SKMEL5 cells treated with 10 µM palbociclib.

Supplementary Figure 5

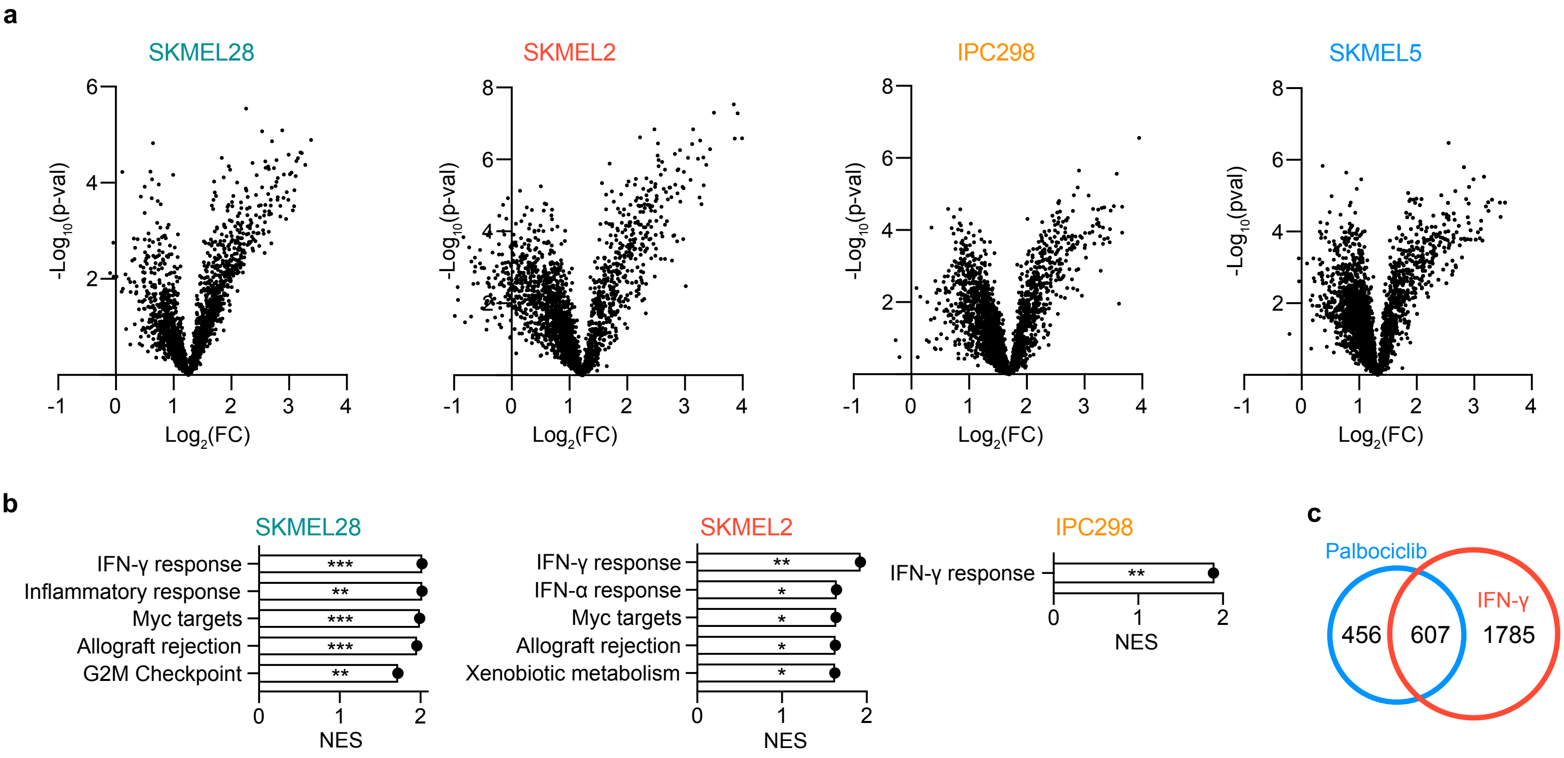

Supplementary Figure 5. a. Volcano plots of the IFN-γ regulated peptides. Data points display the log_2 fold change (FC) of IFN-γ stimulated pMHC abundances over DMSO versus significance (mean adjusted p-value, unpaired two-sided t-test). **b.** Significantly enriched pathways of IFN-γ stimulated cells. Plots represent normalized enrichment score (NES), $q < 0.25$, *$p < 0.05$, **$p < 0.01$, ***$p < 0.001$. Exact p and q-values are reported in the Source Data File. No pathways were negatively enriched. **c.** Venn diagram displaying the number of unique peptides observed in SKMEL5 cells treated with 1 μM palbociclib and/or IFN-γ stimulation.

Supplementary Figure 6

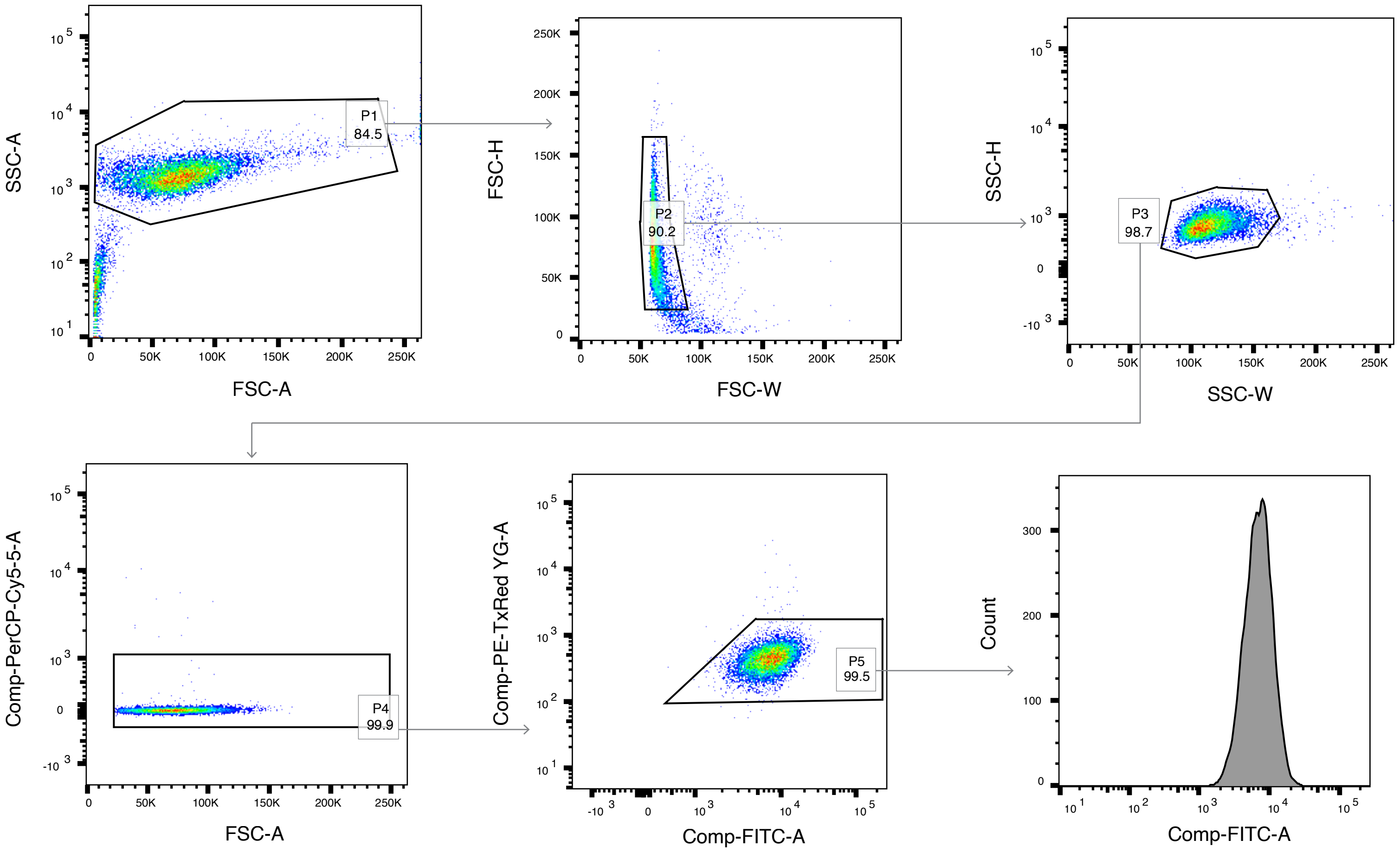

Supplementary Figure 6. Gating strategy for all flow cytometry experiments. FSC-A/SSC-A gating was used to eliminate debris, and doubles were discarded with FSC-W/FSC-H gating followed by SSC-W and SSC-H gating. Living cells were gated by propidium iodide exclusion using FSC-A/PerCP-CY5-5A. FITC positive cells were gated against TX-red, using unstained cells to distinguish FITC positive vs. FITC negative cells.
