# Supplementary material for: Multiplexed relative and absolute quantitative immunopeptidomics reveals MHC I repertoire alterations induced by CDK4/6 inhibition
Source: Nat Commun. 2020 Jun 2;11:2760. doi: 10.1038/s41467-020-16588-9 (PMC7265461; doi:10.1038/s41467-020-16588-9)
Supplement: Supplementary file 2 — Description of Additional Supplementary Files [file 41467_2020_16588_MOESM2_ESM.pdf]

# Description of Additional Supplementary Files

**Supplementary Data 1**. File map & peptide MHC quantification of LF and TMT-labeled MDA-MB-231 analyses

**Supplementary Data 2**. File map and data used for absolute quantification of BCAP31 and DDX5 peptides

**Supplementary Data 3**. File map & peptide MHC quantification of TMT-labeled analyses of melanoma cell lines treated with low/high dose palbociclib treatment

**Supplementary Data 4**. RNA-seq analysis of SKMEL5 cells treated with 1 µM palbociclib

**Supplementary Data 5**. File map & peptide MHC quantification of TMT-labeled analyses in IFN-γ treated melanoma cell lines

**Supplementary Data 6**. Targeted masses for absolute quantification
