# Supplementary material for: Multiplexed relative and absolute quantitative immunopeptidomics reveals MHC I repertoire alterations induced by CDK4/6 inhibition
Source: Nat Commun. 2020 Jun 2;11:2760. doi: 10.1038/s41467-020-16588-9 (PMC7265461; doi:10.1038/s41467-020-16588-9)
Supplement: Supplementary file 9 — Reporting Summary [file 41467_2020_16588_MOESM9_ESM.pdf]

nature research

Corresponding author(s): Forest White

Last updated by author(s): 4/30/2020

Reporting Summary

Nature Research wishes to improve the reproducibility of the work that we publish. This form provides structure for consistency and transparency in reporting. For further information on Nature Research policies, see Authors & Referees and the Editorial Policy Checklist .

Statistics

For all statistical analyses, confirm that the following items are present in the figure legend, table legend, main text, or Methods section.

n/a	Confirmed	
☐	☒	The exact sample size (*n*) for each experimental group/condition, given as a discrete number and unit of measurement
☐	☒	A statement on whether measurements were taken from distinct samples or whether the same sample was measured repeatedly
☐	☒	The statistical test(s) used AND whether they are one- or two-sided<br>*Only common tests should be described solely by name; describe more complex techniques in the Methods section.*
☐	☒	A description of all covariates tested
☐	☒	A description of any assumptions or corrections, such as tests of normality and adjustment for multiple comparisons
☐	☒	A full description of the statistical parameters including central tendency (e.g. means) or other basic estimates (e.g. regression coefficient) AND variation (e.g. standard deviation) or associated estimates of uncertainty (e.g. confidence intervals)
☐	☒	For null hypothesis testing, the test statistic (e.g. *F*, *t*, *r*) with confidence intervals, effect sizes, degrees of freedom and *P* value noted<br>*Give P values as exact values whenever suitable.*
☒	☐	For Bayesian analysis, information on the choice of priors and Markov chain Monte Carlo settings
☒	☐	For hierarchical and complex designs, identification of the appropriate level for tests and full reporting of outcomes
☒	☐	Estimates of effect sizes (e.g. Cohen's *d*, Pearson's *r*), indicating how they were calculated

Our web collection on statistics for biologists contains articles on many of the points above.

Software and code

Policy information about availability of computer code

Data collection	Mass spectrometry data was acquired using Thermo Fisher Scientific Xcalibur version 2.9.0.2923.<br>Phenotypic assays and ELISA results were acquired using Tecan icontrol version 1.7.1.12.<br>IncuCyte data was acquired using IncuCyte Zoom version 6.2.9200.0.<br>Flow cytometry data was acquired using BD FACSDiva version 8.0.<br>RNA-seq data was sequenced using HiSeq Control Software: version 2.2.58, RTA: version 1.18.64.
Data analysis	Flow cytometry data was analyzed using FlowJo version 10.6.1.<br>Mass spectrometry data was searched using Proteome Discoverer version 2.2 and Mascot version 2.4 and analyzed using Proteome Discoverer 2.2 and Skyline version 19.1.0.193, Matlab version R2019b, and Microsoft Excel version 16.34.<br>Pathway enrichment analyses was performed using GSEA 4.0.3 and STRING version 11.<br>Peptide binding affinity of 9mers was predicted using NetMHCpan-4.0.<br>IncuCyte data was analyzed using IncuCyte Zoom version 6.2.9200.0.<br>Data was visualized using GraphPad Prism version 8.4.1.<br>RNAseq reads were aligned to the human transcriptome prepared with the hg38 primary assembly and the Ensembl version 95 annotation using STAR version 2.5.3a. Gene expression was summarized with RSEM version 1.3.0 and SAMtools version 1.3. Differential expression analysis was performed with DESeq2 version 1.24.0 running under R version 3.6.0 with normal log fold change shrinkage. The resulting data were parsed and assembled using Tibco Spotfire Analyst version 7.11.1.

For manuscripts utilizing custom algorithms or software that are central to the research but not yet described in published literature, software must be made available to editors/reviewers. We strongly encourage code deposition in a community repository (e.g. GitHub). See the Nature Research guidelines for submitting code & software for further information.

Data

Policy information about availability of data

All manuscripts must include a data availability statement. This statement should provide the following information, where applicable:

- Accession codes, unique identifiers, or web links for publicly available datasets
- A list of figures that have associated raw data
- A description of any restrictions on data availability

RNA-sequencing data have been deposited into the NCBI Gene Expression Omnibus (GSE144373). The mass spectrometry proteomics data have been deposited to the ProteomeXchange Consortium via the PRIDE partner repository with the dataset identifier PXD017407. Analyzed mass spectrometry immunopeptidomics data from analyses in Figure 2, Figure 3, Figures 4/5, and Figure 6 is available in Supplementary Tables 1, 2, 3, and 4 respectively. File maps linking files to corresponding datasets in the manuscript is available in Supplementary Tables 1-3, 5. Analyzed RNA-seq data is available in Supplementary Table 3. The list of targeted masses used in Figure 3 for absolute quantification of peptide MHCs is listed in Supplementary Table 6. The source data underlining Figs. 2d-h, 3a, 3c-d, 4a, 4d-e, 4h, 5a, 5d-g, 6b-d and Supplementary Figs. 1g, 3b, 3d-e, 3j, and 5a-b are provided as a Source Data File.

Field-specific reporting

Please select the one below that is the best fit for your research. If you are not sure, read the appropriate sections before making your selection.

☒ Life sciences ☐ Behavioural & social sciences ☐ Ecological, evolutionary & environmental sciences

For a reference copy of the document with all sections, see nature.com/documents/nr-reporting-summary-flat.pdf

Life sciences study design

All studies must disclose on these points even when the disclosure is negative.

Sample size	Sample sizes for quantitative mass spectrometry experiments were chosen as n=3 for each condition to assess statistical significance within the capacity of TMT 6-plex isobaric mass tag labeling reagent. For RNA-sequencing analysis, n=3 was also chosen to assess statistical significance between conditions, with a matching number of samples to the MS analysis. Phenotype measurements were similarly performed with at least n=3 experimental replicates to determine significance. In each case, the number of samples was chosen to allow for significance values to be calculated while minimizing the reagent and instrument time costs.
Data exclusions	For mass spectrometry experiments, peptides were filtered according to the listed criteria listed in the methods to identify only high confidence peptide spectrum matches (PSMs), while excluding peptides that were not likely to be MHC binders (peptides less than 8 amino acids or greater 15). These criteria were pre-established prior to data analysis. We also excluded hipMHC PSMs that had an average reporter ion intensity outside 10-fold of the interquartile range of endogenous PSM reporter ion intensities. This exclusion was established when we observed a drift in correction factors when PSM TMT intensities were well beyond endogenous levels in SKMEL5 cells +/- 1uM CDK4/6i. After this finding, this exclusion was applied to all subsequent analyses.
Replication	Mass spectrometry findings were replicated within multiplexed analyses which utilized 3 biological replicates of samples. Phenotypic assays of drug sensitivity and flow cytometry experiments were repeated two to three times with similar similar results, results presented are representative.
Randomization	Cell culture experiments were seeded from the same initial stock of cells, therefore randomization in treatment groups among cell culture dishes was not necessary. TMT labeling was not randomized (untreated samples were labeled with TMT126-128 and treated samples were labeled with TMT129-131 for consistency and interpretability), but any disparities related to a specific TMT channel was corrected for with hipMHC normalization.
Blinding	Investigators were not blinded to group allocations within analyses, though is not relevant for this study given the automated nature of data collection and analyses performed. For mass spectrometry data analysis, filtering criteria were established prior to analysis. Similarly, RNA-sequencing analysis follows a standard protocol established prior to data collection. No blinding was necessary analyzing the phenotype data, as all data was collected using plate readers and automated imaging systems. For flow cytometry analysis, the same set of gates defined using an untreated control sample of the same cell line was applied to all samples within that cell line and treatment conditions.

Reporting for specific materials, systems and methods

We require information from authors about some types of materials, experimental systems and methods used in many studies. Here, indicate whether each material, system or method listed is relevant to your study. If you are not sure if a list item applies to your research, read the appropriate section before selecting a response.

Materials & experimental systems

n/a	Involved in the study	
☐	☒	Antibodies
☐	☒	Eukaryotic cell lines
☒	☐	Palaeontology
☒	☐	Animals and other organisms
☒	☐	Human research participants
☒	☐	Clinical data

Methods

n/a	Involved in the study	
☒	☐	ChIP-seq
☐	☒	Flow cytometry
☒	☐	MRI-based neuroimaging

Antibodies

Antibodies used

Biolegend: Alexa Fluor 488 HLA-A, B, C, clone W6/32, [cat # 311413], Lot: B256489, Alexa Fluor 488 anti-H2A.X Phospho (Ser139), clone 2F3, [cat # 613406], Lot: 271052. BioXcell: inVivoMAb anti-human MHC Class I (HLA-A, HLA-B, HLA-C), clone W6/32, [cat # BE0079] Lots: 680817D1 & 457417S1.

Validation

Anti-human MHC Class I (HLA-A, HLA-B, HLA-C), clone W6/32 is a commonly used pan-specific antibody for MHC Class I alleles that recognizes the residues in the N terminus of β 2-microglobulin molecule, validated previously (Shields MJ. Ribaudo RK. 1998. Tissue Antigens. 51(5):567-70, Stern P, et al. 1987. J. Immunol. 138:1088).

W6/32 is also validated in its specificity for Class-I HLA alleles in the manuscript, as a majority of peptides purified with W6/32 using immunoprecipitation are predicted to be binders of a cell line's known allelic profile. The fluorophore conjugated antibody is quality tested via Biolegend. H2A.X Phospho (Ser139) antibody, recognizing ser139 phosphorylated H2A-X, which occurs after double stranded breaks. This antibody is quality tested via Biolegend, and has cited in the literature (Jha JC, et al. 2013. J. Virol. 87:5255) for this application.

Eukaryotic cell lines

Policy information about cell lines

Cell line source(s)

SKMEL5, SKMEL28, and MDA-MB-231 cell lines were purchased from ATCC, IPC298 and SKMEL2 were obtained from Array BioPharma.

Authentication

Cell lines obtained directly from ATCC have been authenticated, using morphology, karyotyping, and PCR based approaches. Cell lines obtained from Array BioPharma were not authenticated upon receival.

Mycoplasma contamination

Cells lines tested negative for mycoplasma contamination both after thawing and in the passage prior to sample generation. Samples were confirmed negative using MycoAlert (Lonza) Mycoplasma Detection Kit.

Commonly misidentified lines
(See ICLAC register)

None

Flow Cytometry

Plots

Confirm that:

- ☒ The axis labels state the marker and fluorochrome used (e.g. CD4-FITC).
- ☒ The axis scales are clearly visible. Include numbers along axes only for bottom left plot of group (a 'group' is an analysis of identical markers).
- ☐ All plots are contour plots with outliers or pseudocolor plots.
- ☐ A numerical value for number of cells or percentage (with statistics) is provided.

Methodology

Sample preparation

For HLA expression analysis of live cells by flow cytometry, cells were lifted with 0.05% Trypsin-EDTA and 10^6 cells/mL were spun at 300 g for 3 minutes, washed with ice cold PBS supplemented with 1% FBS and 0.1% sodium azide (flow buffer) and incubated with fluorophore-conjugated antibody at 0.5ug/mL in flow buffer for 30 minutes on ice. After incubation, cells were washed again, and resuspended in flow buffer plus 5 µL of propidium iodide staining solution (10 µg/mL, Invitrogen) per sample. To measure H2A.X levels, 10^6 cells were fixed with 70% ethanol for 2 hours at -20C. Cells were then washed 2x with flow buffer, and and incubated with fluorophore-conjugated antibody at 0.5ug/mL in flow buffer for 30 minutes on ice. Cells were then washed with flow buffer and analyzed.

Instrument

Analyses were performed on an BD Biosciences LSR II.

Software

Flow cytometry data was acquired using BD FACSDiva version 8.0 and analyzed using FlowJo version 10.6.2.

Cell population abundance	10,000 events (gated) were collected per sample. Dead cells in live cell analysis were excluded.
Gating strategy	FSC-A/SSC-A gating was used to eliminate debris, and doubles were discarded with FSC-W/FSC-H gating followed by SSC-W and SSC-H gating. Living cells were gated by propidium iodide exclusion using FSC-A/PerCP-CY5-5A. FITC positive cells were gated against TX-red, using unstained cells to distinguish FITC positive vs. FITC negative cells.

☒ Tick this box to confirm that a figure exemplifying the gating strategy is provided in the Supplementary Information.
